# Non-Markovian Electron Transfer in Ligand–Receptor Complexes: Insights from Non-Gaussian Anharmonic Baths

**DOI:** 10.1021/acs.jpcb.6c00165

**Published:** 2026-04-15

**Authors:** Muhammad Waqas Haseeb, Mohamad Toutounji

**Affiliations:** † Department of Physics, 11239 United Arab Emirates University, Al-Ain 15551, UAE; ‡ Department of Chemistry, United Arab Emirates University, Al-Ain 15551, UAE

## Abstract

Electron transfer (ET) in protein receptor–ligand complexes is governed by environmental structure, memory, and fluctuation statistics. We investigate ET dynamics within a non-Markovian open-quantum-systems framework using a non-Markovian stochastic Schrödinger equation (NMSSE), contrasting the conventional *harmonic* (Gaussian) bath approximation with an *anharmonic*, non-Gaussian environment modeled by discrete Poisson (shot-noise) events. The model consists of a two-state donor–acceptor dimer coupled to a discrete vibrational mode and embedded in a structured protein–membrane environment. To represent anharmonicity beyond harmonic-bath theory, we introduce a finite-memory shot-noise description at the level of the second cumulant that implements instantaneous kicks on the coupled electronic–vibrational manifold. Ensemble-averaged trajectory simulations yield populations and coherences across broad parameter ranges. Three robust regimes emerge: (i) a *weakly anharmonic* regime, where many small events per correlation time render the compound-Poisson bath effectively Gaussian and harmonic, and non-Gaussian predictions are quantitatively close; (ii) an *intermediate anharmonic* regime, where intermittency and higher-order statistics become dynamically relevant, enhancing ET and qualitatively reshaping population and coherence dynamics, particularly at weak electronic coupling; and (iii) a *strongly anharmonic* sparse-event regime, where impulsive events drive pronounced, irregular energy exchange and the largest deviations from harmonic-bath behavior. These results delineate when harmonic approximations are sufficient and when explicit anharmonic, non-Gaussian bath models are required for faithful ET dynamics in biomolecular environments.

## Graphical abstract

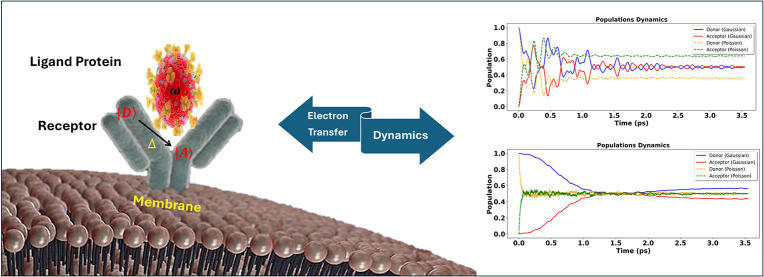

## 1 Introduction

Electron transfer (ET) in proteins is a canonical open-quantum-systems problem: the electronic degrees of freedom evolve under continuous influence from a structured, dissipative environment comprising intramolecular vibrations, solvent fluctuations, and membrane modes.^1^ In such situations, environmental memory and structure frequently invalidate the simplifying assumptions that underlie Markovian master equations, motivating non-Markovian descriptions that retain time correlations and backflow of information.^2–5^ Beyond purely theoretical appeal, these effects have practical implications: environmental structure and noise statistics control rates, coherence lifetimes, and even qualitative transport pathways in proteinaceous media. Growing evidence suggests that quantum transport fundamentally impacts key biological processes such as photosynthesis and protein electron transfer.^6–10^ While semiclassical theories like Marcus–Jortner are useful for understanding fundamental concepts like activation and reorganization in chemical reactions, they are limited by their underlying assumptions. These theories generally require the surrounding environment to behave like a simple harmonic oscillator (a ″bath″), assume weak interaction with the reaction, and can only model fast environmental changes.^11^ To accurately model systems where semiclassical assumptions fail specifically in low-frequency, highly dissipative environments, or those with complex spectral features in in the energy landscape, a dynamical, fully quantum approach is essential to use.^12^

Vibration-assisted electron transfer (VA-ET) is critical because discrete molecular modes can tune both selectivity and efficiency of charge transport. When specific vibrational frequencies match electronic energy gaps, they promote resonant coupling that activates or markedly amplifies otherwise weak ET pathways.^13,14^ This perspective elevates environmental structure to a primary determinant of ET and motivates a central modeling question: how strongly do the *noise statistics* Gaussian versus non-Gaussian, continuous fluctuations versus discrete jump events govern ET dynamics in realistic protein settings?

A central question is whether the donor–acceptor ET dynamics considered here could be treated nearly classically and/or within a Markovian approximation. In our model parametrization the relevant energy scales are comparable to, or exceed, thermal energy. In particular, the discrete vibrational mode has *ℏω* = 0.1487 eV while *k*_B_ *T* = 0.025 eV, so *ℏω*/*k*_B_ *T* ≃ 5.95, implying strongly quantized vibrational dynamics (the thermal occupation *n*_th_ = [*e* ^ω/T^ – 1]^−1^ ≈ 2.6 × 10^–3^). Likewise, the intrinsic electronic frequency $\Omega =\sqrt{\epsilon^{2}+\Delta^{2}}$ lies in the range Ω ≃ 0.149–0.179 eV for ϵ = 0.1487 eV and Δ∈ [10^–4^,10^–1^] eV, giving Ω/*k*_B_ *T* ≃ 5.95–7.2. These ratios place the dynamics outside a purely classical limit in which vibronic quantization and coherence can be neglected. Non-Markovianity is motivated by the bath memory scale relative to τ_sys_ ∼ Ω^–1^. For the structured bath used in our non-Markovian simulations, the line width parameter Γ = 0.005 eV sets a correlation time of order τ_*c*_ ∼ Γ^–1^ ≈ 200 (in units with *ℏ* = 1), whereas τ_sys_ ∼ Ω^–1^ ≈ 5.6–6.7, so that τ_*c*_/τ_sys_ ∼ 30–36. Equivalently, the dimensionless non-Markovianity measure η_NM_≡Ωτ_*c*_ ∼ Ω/Γ is η_NM_ ≫ 1, indicating that bath correlations persist on time scales comparable to, or longer than, the intrinsic ET dynamics and that time-local Markovian reductions are not expected to be quantitatively reliable.

The traditional representation of the environment as a bath of harmonic oscillators yields Gaussian fluctuations by construction and has underpinned decades of progress due to its analytic tractability and clear connection to spectral densities.^1^ However, biomolecular surroundings can exhibit discrete, intermittent, or strongly anharmonic interactions (two-state subunits, localized bonds, rare but large kicks) that are more naturally modeled by non-Gaussian processes.^15–17^ The Lévi–Ito decomposition further justifies this generalization by showing that white noise can be decomposed into Gaussian and Poisson components, suggesting that jump processes form a natural complement to Gaussian models when discrete events matter.^18,19^ From a transport standpoint, such jump-like fluctuations can change not only rates but also the qualitative time dependence of populations, especially when the bath correlation time is finite and the coupling is not perturbatively small.

Among several routes to non-Markovian dynamics, the non-Markovian quantum state diffusion (NMQSD) formalism and its stochastic Schrödinger (NMSSE) realizations provide a trajectory-based framework that links dynamics directly to bath correlation functions.^20,21^ NMQSD enables the inclusion of structured spectral densities as well as systematic perturbative treatments of memory kernels, and can be extended to incorporate non-Gaussian fluctuations via suitably constructed noise processes or jump operators.^22^ This combination is attractive for biomolecular ET because it preserves microscopic interpretability (via spectral densities and correlation functions) while allowing one to interrogate how discrete environmental noise statistics alter dynamical observables such as population transfer and coherence.

In this work we use a minimal receptor–ligand donor–acceptor dimer, coupled to a discrete vibrational mode and embedded in a structured protein–membrane environment, as a model platform to investigate how environmental memory and bath anharmonicity reshape ET dynamics. Figure 1 shows a simplified illustration of the model. Our aim is methodological rather than system-specific: we do *not* propose a biochemical mechanism for any particular complex. Instead, we address the general dynamical question: *when is the harmonic (Gaussian) bath approximation sufficient, and when do anharmonic, jump-like (non-Gaussian) environmental fluctuations qualitatively modify ET?* The Hamiltonian adopts a spin–boson construction in which a two-level electronic subsystem (donor/acceptor) is coupled to selected vibrational coordinates and to an environmental reservoir, consistent with established formulations of protein ET and vibronic coupling.^23–25^

**Figure 1:**
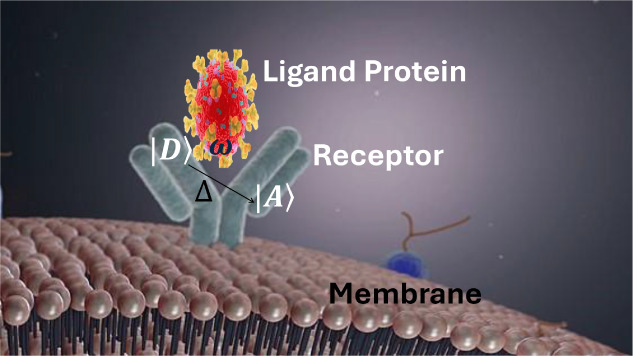
Schematic of intraprotein electron transfer (ET) initiated upon engagement of the host receptor with the ligand protein. The simplified graphical model highlights putative donor and acceptor sites and the ET pathways that bridge the Ligand–Receptor interface.

We contrast two environmental descriptions within a unified NMSSE framework: (i) a *harmonic* (Gaussian) bath specified by a structured spectral density, which fully determines the dynamics through its two-point correlation function, and (ii) an *anharmonic*, non-Gaussian environment modeled as a compound Poisson (shot-noise) process with discrete events. In the latter case we retain finite memory by introducing an effective colored shot-noise kernel at the second-cumulant level and, additionally, include unitary jump operators that implement instantaneous kicks on the coupled electronic–vibrational manifold. Using ensemble-averaged trajectory simulations, we map ET behavior across electronic coupling strengths, bath memory times, and event-rate regimes. Three robust regimes emerge: (a) a weakly anharmonic regime (many small events per correlation time) where Gaussian and Poisson descriptions largely agree, (b) an intermediate/strongly anharmonic regime (sparse, intermittent events) where non-Gaussian statistics enhance ET and qualitatively reshape population and coherence dynamics even at weak electronic coupling, and (c) a strong electronic-coupling regime where sensitivity to bath statistics diminishes and the two descriptions converge. These findings clarify when harmonic modeling is adequate and when explicit anharmonic, non-Gaussian bath descriptions are required for faithful ET dynamics in complex biomolecular environments.

While our focus is methodological, the results suggest practical routes toward *system-specific* studies: spectral densities and correlation times can be constrained by vibrational spectroscopy (e.g., Raman/2D techniques) and related to model parameters,^26,27^ and trajectory-level predictions of ET dynamics could be probed via quantum biological electron-tunneling (QBET) measurements in suitably engineered constructs.^28^ Incorporating such data would enable targeted inversion of bath parameters (e.g., memory times, jump rates) and hypothesis testing for concrete protein systems.

Section 2 (*Theory and Methods*) develops the NMSSE formalism used here, defines the Gaussian and Poisson baths and jump operators, and specifies the structured spectral densities and finite-temperature correlation functions.^29^ Section 3 (*Results and Discussion*) presents population dynamics and ET Probabilities maps across coupling and anharmonicity regimes. Section 4 (*Conclusions*) summarizes the implications for modeling ET in proteins and outlines next steps toward system-specific parametrization and experimental validation.

## 2 Theory and Methods

Quantum systems within biological environments rarely function in isolation; instead, they are constantly influenced by numerous surrounding bio molecular entities that contribute to a dynamic and noisy background. To model these systems faithfully, one must explicitly incorporate their environmental couplings. The open-quantum-systems (OQS) framework provides a systematic route to do so^1^ by specifying the system Hamiltonian, the bath Hamiltonian, and the interaction terms that mediate energy and information exchange between them.

A standard analytical route to open-system ET dynamics is to derive quantum master equations for the reduced density operator by tracing out environmental degrees of freedom. Prominent examples include the Nakajima–Zwanzig projection-operator formalism,^2^ Redfield theory,^3^ completely positive Lindblad generators,^4^ the Hu–Paz–Zhang model,^30^ and broader classes of time-local non-Markovian master equations.^5^ While these approaches are powerful, their practical applicability is often tied to specific assumptions (e.g., weak system–bath coupling, short bath memory, or Gaussian statistics) and can become cumbersome when one wishes to combine *structured non-Markovian memory* with *intermittent, non-Gaussian environmental events.* ^31^

Semiclassical rate theories (including Fermi’s Golden Rule estimates in the weak-coupling limit) provide useful intuition, but they can break down in low-frequency or strongly dissipative environments and when vibronic structure is dynamically relevant. For example, polaron-based master equations demonstrate that strong dissipation can qualitatively modify transport and switching behavior,^12^ highlighting that environmental structure can control dynamics beyond simple Markovian or harmonic-bath pictures. Motivated by these limitations, we adopt a trajectory-based non-Markovian stochastic Schrödinger equation (NMSSE) framework, which incorporates structured bath memory directly through a correlation kernel and its associated time-dependent coefficients, while allowing non-Gaussian intermittency to be added at the trajectory level via compound-Poisson (shot-noise) events. This combination provides a scalable and conceptually transparent route to assess, within a unified dynamical framework, when harmonic/Gaussian bath models are adequate and when explicitly non-Gaussian, jump-like environmental statistics become essential.

Several complementary lines of work have examined when the harmonic (Gaussian) bath approximation is reliable and how one can go beyond it. Foundational treatments of dissipative quantum dynamics and the spin–boson model establish the harmonic-bath baseline and its regime of validity, including influence-functional formulations and their common reductions.^32–35^ For strongly coupled and/or non-Markovian structured environments, numerically controlled “exact” approaches such as the hierarchical equations of motion (HEOM) and related reduced-hierarchy formulations provide benchmark-quality dynamics at the cost of steep scaling with bath structure and hierarchy depth.^36–38^ Wave function/trajectory-based exact methods for structured non-Markovian baths, including non-Markovian quantum state diffusion (NMQSD) and the hierarchy of pure states (HOPS), provide an alternative route that retains microscopic interpretability while improving numerical scaling in many settings.^39–40^ Beyond Gaussian statistics, shot-noise and jump-process environments have long been studied in transport and open-system contexts,^15^ and recent work has developed master-equation descriptions for Poissonian (white) non-Gaussian baths.^31^ The present work builds on this literature by combining (i) finite-memory non-Markovian dynamics parametrized by a structured kernel with (ii) explicit intermittent (compound-Poisson) event statistics at the trajectory level, enabling a controlled comparison of harmonic versus anharmonic/non-Gaussian bath descriptions across event-rate and coupling regimes.

We also contextualize our approach relative to established non-Markovian benchmark methods. Hierarchical equations of motion (HEOM) and closely related reduced-hierarchy formulations provide numerically controlled, essentially exact dynamics for a wide class of Gaussian environments, including structured spectral densities and finite temperature, and are therefore widely used as accuracy benchmarks.^36–38^ Their main limitation is computational scaling: the number of auxiliary density operators grows rapidly with decreasing temperature, increasing bath correlation time (more exponentials required to represent the kernel), stronger system–bath coupling, and larger system Hilbert spaces, which can make parameter scans expensive in structured environments. Trajectory-based exact methods such as the hierarchy of pure states (HOPS) provide an alternative exact route for Gaussian baths, with scaling that can be favorable in some structured cases but still requiring increasing hierarchy depth as memory effects strengthen.^40^ Polaron and variational-polaron master equations offer complementary approximations that can remain accurate at stronger coupling by partially resumming system–bath interactions, but they typically rely on specific assumptions about bath structure and do not directly capture non-Gaussian intermittency.^12^ In contrast, the NMSSE framework employed here is naturally formulated at the trajectory level and incorporates bath memory through time-dependent coefficients derived from the correlation kernel. Crucially, its perturbative time-local expansion can be truncated at low order (here through *g*_0_ and *g*_1_) to yield a scalable model for structured non-Markovian dynamics, while still retaining the leading memory corrections; this makes broad parameter mapping feasible even as memory effects become significant. Our additional non-Gaussian extension is implemented through explicit compound-Poisson event statistics (jump/kick realizations), which is straightforward to incorporate in a trajectory picture but is not directly accommodated in standard Gaussian-bath HEOM/Redfield-type frameworks. Together, these considerations motivate NMSSE as a computationally efficient and flexible platform for identifying when harmonic-bath approximations are adequate and when finite-memory, non-Gaussian intermittency must be treated explicitly.

In the following, we consider a quantum system coupled to a bosonic bath, described by the Hamiltonian^21^ (with *ℏ* = 1):

1 $$H=H_{S}+\underset{k}{\sum }\left(g_{k}Lb_{k}^{†}+g_{k}^{*}L^{†}b_{k}\right)+\underset{k}{\sum }\omega_{k}b_{k}^{†}b_{k}$$

where *H*_S_ is the system Hamiltonian, *L* is the system coupling operator, *b*_*k*_ annihilates the *k*-th bath mode of frequency ω_*k*_, and *g*_*k*_ denotes the coupling strength.^20^ In the coherent-state representation underlying non-Markovian quantum state diffusion (NMQSD), the bath influence enters through a complex Gaussian noise process $z_{t}^{*}$ with

2 $$M\left[z_{t}\right]=0,,M\left[z_{t}z_{s}^{*}\right]=\alpha_{G}\left(t,s\right)$$

where M[·] denotes the ensemble average and the bath kernel is

3 $$\alpha_{G}\left(t,s\right)=\underset{k}{\sum }|g_{k}{|}^{2}e^{-i\omega_{k}(t-s)}$$

(at *T* = 0; finite-temperature expressions are obtained by the standard coth­(ω/2*k*_B_ *T*) modification). Because the bath is harmonic, the noise is Gaussian and is fully characterized by the two-point function α_*G*_(*t*,*s*).

To go beyond the harmonic approximation and capture discrete, intermittent environmental events (e.g., barrier-crossings, rare conformational switches, and impulsive force bursts that are natural in strongly anharmonic potentials such as Morse surfaces), we model the bath fluctuations by a *compound Poisson* (shot-noise) process. Importantly, Poissonian baths are intrinsically *non-Gaussian*: higher-order cumulants do not vanish, and therefore the statistics are not fully determined by a two-point correlator alone. Nevertheless, in the time-local perturbative NMSSE used below, the bath enters through the second cumulant, and hence through an effective two-time kernel α_*P*_(*t*,*s*). We make this second-cumulant closure explicit and later supplement it with trajectory-level jump realizations when needed.

Let *N*(*dt*,*da*) be a Poisson random measure (PRM) on time and a “mark” variable *a* (the kick amplitude) with intensity measure

4 $$E\left[N\left(dt,da\right)\right]=\nu \left(da\right)dt,,\nu \left(da\right)=\lambda_{p}p\left(a\right)da$$

where λ_*p*_ is the event rate and *p*(*a*) is a normalized amplitude distribution. To work with a zero-mean process we introduce the compensated PRM

5 Ñ(dt,da)=N(dt,da)−ν(da)dt

We then define the bath process that drives the system as a *filtered* compound-Poisson noise

6 $$z\left(t\right)=\int_{0}^{t}\int a\phi \left(t-u\right)Ñ\left(du,da\right)$$

where ϕ­(*t*) is a causal response (filter) function encoding the bath memory and characteristic frequencies. This construction captures discrete events (through Poisson arrivals) while retaining finite correlation time (through ϕ), thereby providing a controlled bridge between purely Markovian jump noise and structured non-Markovian environments.

Using the isometry property of compensated Poisson measures, the second moment of the process ([Disp-formula eq6]) is^41,42^

7 $$\alpha_{P}\left(t,s\right)≡E\left[z\left(t\right)z^{*}\left(s\right)\right]=\int_{0}^{\min(t,s)}du\left(\int a^{2}\nu \left(da\right)\right)\phi \left(t-u\right)\phi^{*}\left(s-u\right)$$

For stationary conditions (times long compared to the bath memory), α_*P*_(*t*,*s*) depends only on τ = *t* – *s*, and can be written as

8 $$\alpha_{P}\left(\tau \right)=\left(\int a^{2}\nu \left(da\right)\right)\int_{0}^{\infty }du\phi \left(u+|\tau |\right)\phi^{*}\left(u\right)$$

The prefactor $\int a^{2}\nu \left(da\right)=\lambda_{p}E\left[a^{2}\right]$ is the *second jump moment*. It controls the overall strength of the second-cumulant bath fluctuations that enter the perturbative NMSSE coefficients. The effective kernel α_*P*_(*t*,*s*) in eqs 7 and 8 is used to parametrize bath memory at the *second-cumulant* level and thereby to compute the time-dependent coefficients *g*_0_(*t*) and *g*_1_(*t*) entering the perturbative time-local NMSSE. This does not, by itself, render the bath Gaussian: the underlying compound-Poisson process has nonzero higher cumulants. In our numerical implementation, higher-order statistics enter explicitly at the trajectory level through Poisson-distributed increments/jump events (shot-noise kicks), so that ensemble averages compare Gaussian and non-Gaussian baths under variance-matched conditions. For the compensated compound-Poisson increment *dX* with rate λ_*p*_ and mark distribution *p*(*a*), the cumulants satisfy $\kappa_{n}\left[dX\right]=\lambda_{p}E\left[a^{n}\right]dt$ for *n* ≥ 2 (and E[dX]=0 by compensation), demonstrating non-Gaussianity beyond the second moment.

In the zero-temperature limit, the evolution of the system’s quantum state follows a linear, time-local SSE.

9 $$\frac{\partial }{\partial t}\left|\psi_{z^{*}}\left(t\right)\right\rangle =\left[-iH_{\mathrm{sys}}+Lz_{t}^{*}-L^{†}\mathrm{O̅}\left(t,z^{*}\right)\right]\left|\psi_{z^{*}}\left(t\right)\right\rangle$$

where *O* is an operator ansatz defined by the functional derivative

10 $$\frac{\delta }{\delta z_{s}^{*}}\left|\psi_{z^{*}}\left(t\right)\right\rangle =O\left(t,s,z^{*}\right)\left|\psi_{z^{*}}\left(t\right)\right\rangle$$

and $\mathrm{O̅}\left(t,z^{*}\right)=\int_{0}^{t}\alpha \left(t,s\right)O\left(t,s,z^{*}\right)ds$ .

The system’s reduced density operator ρ_*s*_(*t*) can be calculated through ensemble averages of quantum trajectories.

11 $$\rho_{s}≡{\mathrm{Tr}}_{\mathrm{env}}\left[e^{-iHt}\left|\psi_{0}\right\rangle \langle \psi_{0}|⊗\rho_{\mathrm{env},0}e^{iHt}\right]=M\left[\left|\psi_{t}\left(z\right)\right\rangle \langle \psi_{t}\left(z\right)|\right]$$

The NMQSD approach involves deriving the functional derivative *O* operator, which can be exact for simple models or perturbatively derived for more general systems.

The NMQSD predicated on states with normalization

12 $${\mathrm{\psi ̃}}_{t}\left(z\right)=\frac{\psi_{t}\left(z\right)}{∥\psi_{t}\left(z\right)∥}$$

could be achieved using the Girsanov transformation.^43^

13 $$\frac{d}{dt}{\mathrm{\psi ̃}}_{t}=-iH_{S}{\mathrm{\psi ̃}}_{t}+\left(L-\langle L\rangle_{t}\right){\mathrm{\psi ̃}}_{t}{\mathrm{z̃}}_{t}-\int_{0}^{t}ds\alpha \left(t,s\right)\left\langle \left(L^{†}-\langle L^{†}\rangle_{s}\right)Ô\left(t,s,{\mathrm{z̃}}_{t}\right)-\left(L^{†}-\langle L^{†}\rangle_{s}\right)Ô\left(t,s,{\mathrm{z̃}}_{t}\right)\right\rangle {\mathrm{\psi ̃}}_{t}$$

Where *z̃*_*t*_ is the shifted noise,

14 $${\mathrm{z̃}}_{t}=z_{t}+\int_{0}^{t}ds\alpha \left(t,s\right)\langle L^{†}\rangle_{s}$$

and ⟨*L*⟩_*s*_ = ⟨*ψ̃*_*t*_|*L*|*ψ̃*_*t*_⟩ is the quantum average.

The nonlinear NMSSE can be written in a compact form by introducing Δ_*t*_(*A*) = *A* – ⟨*A*⟩_*t*_

15 $$\frac{d}{dt}{\mathrm{\psi ̃}}_{t}=-iH_{S}{\mathrm{\psi ̃}}_{t}+\Delta_{t}\left(L\right){\mathrm{\psi ̃}}_{t}z_{t}^{*}-\Delta_{t}\left(L^{†}\right)\mathrm{O̅}\left(t,\mathrm{z̃}\right){\mathrm{\psi ̃}}_{t}+\Delta_{t}\left(L^{†}\right)\mathrm{O̅}(t,\mathrm{z̃}{)}_{t}{\mathrm{\psi ̃}}_{t}$$

Where

16 $$\mathrm{O̅}\left(t,z\right)=\int_{0}^{t}ds\alpha \left(t,s\right)Ô\left(t,s,z\right)$$

The eq 13 or 15 is fundamental NMSSE and the perturbative treatment starts with this equation. Applying the formal Perturbation theory on operator *Ô*(*t*, *s*, *z*) using a series expansion in powers of (*t* – *s*).^20^

17 $$\frac{d}{dt}{\mathrm{\psi ̃}}_{t}=-iH_{Sys}{\mathrm{\psi ̃}}_{t}+\Delta_{t}\left(L\right){\mathrm{z̃}}_{t}-g_{0}\left(t\right)\left(\left(\Delta_{t}\left(L^{†}\right)L-\langle \Delta_{t}\left(L^{†}\right)L\rangle_{t}\right)\right){\mathrm{\psi ̃}}_{t}+ig_{1}\left(t\right)\left(\Delta_{t}\left(L^{†}\right)\left[H,L\right]-\langle \Delta_{t}\left(L^{†}\right)\left[H,L\right]\rangle_{t}\right)){\mathrm{\psi ̃}}_{t}+g_{2}\left(t\right)\left(\left(\Delta_{t}\left(L^{†}\right)\left[L^{†},L\right]L-\langle \Delta_{t}\left(L^{†}\right)\left[L^{†},L\right]\rangle_{t}\right)\right){\mathrm{\psi ̃}}_{t}$$

Within NMQSD, the first order corrections governed by the system frequency ω and the relaxation rate Γ. These corrections is confirmed when the environmental correlation time is finite yet remains shorter than the intrinsic time scales of the system, thereby validating the use of the expanded NMQSD framework. As the correlation time approaches zero, the system’s dynamics simplify to the Markovian limit, wherein only the zeroth-order term persists. This boundary explicitly defines the conditions for non-Markovian versus Markovian quantum behavior.

The time-dependent coefficients *g*_*i*_(*t*) are determined by the environmental correlation function α_*(G,p)*_(*t*,*s*):

18 $$g_{0}\left(t\right)=\int_{0}^{t}\alpha_{i}\left(t,s\right)ds$$

19 $$g_{1}\left(t\right)=\int_{0}^{t}\alpha_{i}\left(t,s\right)\left(t-s\right)ds$$

20 $$g_{2}\left(t\right)=\int_{0}^{t}du\int_{0}^{s}\alpha_{i}\left(t,s\right)\alpha_{i}\left(s,u\right)\left(t-s\right)ds$$

This study extends the NMQSD/NMSSE framework to investigate how electron tunneling in a generic receptor–ligand donor–acceptor platform is shaped by vibrational coupling and by the statistical character of environmental fluctuations. The system is modeled within a spin–boson formulation, in which a two-level electronic dimer (donor/acceptor) is coupled to a discrete vibrational coordinate and embedded in a structured environment that may exhibit finite memory.^23,24^ Motivated by broader ideas of vibration-assisted tunneling in biomolecular settings,^13^ we analyze how resonant vibrational structure and non-Gaussian intermittency in the surrounding bath can modulate ET probabilities and the associated population and coherence dynamics.

The receptor is modeled as a two-level dimer with the Hamiltonian:

21 $$H_{R}=\frac{1}{2}\epsilon \sigma_{z}+\frac{1}{2}\Delta \sigma_{x}$$

where ϵ = ϵ_*D*_ – ϵ_*A*_ is the energy difference between donor and acceptor sites, and Δ is the tunneling coupling. For an isolated dimer with ϵ = 0, the maximum transition probability is 1.^25^

22 $$\mathrm{Max}\left[P_{D\to A}\left(t\right)\right]=\frac{\Delta^{2}}{\Delta^{2}+\epsilon^{2}}$$

The ligand is represented as a harmonic oscillator, and its interaction with the receptor couples the protein’s vibrational modes to the electronic states of the receptor:

23 $$H_{P}=\omega \left(b^{†}b+1/2\right)$$

24 $$H_{R-P}=\sigma_{z}\underset{i}{\sum }\omega_{i}\gamma_{i}\left(b_{i}+b_{i}^{†}\right)$$

The total system Hamiltonian, combining the receptor, ligand protein and their interaction, is

25 $$H_{S}=\frac{1}{2}\epsilon \sigma_{z}+\frac{1}{2}\Delta \sigma_{x}+\omega \left(b^{†}b+1/2\right)+\sigma_{z}\underset{i}{\sum }\omega_{i}\gamma_{i}\left(b_{i}+b_{i}^{†}\right)$$

The surrounding biological membrane is modeled as an anharmonic environment of Morse oscillators, whose interaction with the receptor is described by

26 $${Ĥ}_{E}^{\mathrm{anharm}}=\overset{N}{\underset{n=1}{\sum }}\overset{\Lambda }{\underset{j=1}{\sum }}D_{nj}\left(e^{-2\alpha_{nj}x_{nj}}-2e^{-\alpha_{nj}x_{nj}}\right)$$

27 $${Ĥ}_{\mathrm{R‐E}}^{\mathrm{anharm}}=\overset{N}{\underset{n=1}{\sum }}\overset{\Lambda }{\underset{j=1}{\sum }}\gamma_{nj}\left(1-e^{-\alpha_{nj}x_{nj}}\right){\mathrm{\sigma ̂}}_{z}$$

Here, *D*_*nj*_ = ω_*nj*_ η^2^ and $\alpha_{nj}=\sqrt{\omega_{nj}\eta }$ are the depth and width of the Morse potential, with η being the number of bound states. We model the anharmonic membrane environment as a *compound Poisson (shot-noise) bath*, where discrete perturbations occur at random times with rate λ_*p*_ and with random amplitudes (marks). Because Poisson noise is non-Gaussian, higher cumulants are generally nonzero; however, to incorporate finite memory within our perturbative NMSSE approach we represent the bath at the second-cumulant level using an effective colored kernel α_*P*_(τ) = *A*_0_ *e* ^–κ|τ|^cos­(ω_*P*_τ), with correlation time τ_*c*_ = κ^–1^ and amplitude $A_{0}\propto \lambda_{p}E\left[a^{2}\right]/\kappa$ .

Although we present results for a minimal donor–acceptor dimer coupled to a single discrete vibrational coordinate, the main conclusions are organized in terms of dimensionless control parameters that apply broadly to receptor–ligand ET models within the spin–boson class. The first control parameter is the *non-Markovianity* measure $\eta_{\mathrm{NM}}≡\Omega \tau_{c},where\Omega =\sqrt{\epsilon^{2}+\Delta^{2}}$ , which compares the intrinsic electronic time scale τ_sys_ ∼ Ω^–1^ to the bath correlation time τ_*c*_. The second is the *event-per-memory* parameter Λ≡λ_*p*_τ_*c*_, which quantifies the expected number of discrete environmental events occurring within one correlation time and thereby controls the crossover from effectively Gaussian behavior (Λ ≫ 1) to intermittency-dominated non-Gaussian dynamics (Λ ≪ 1). Additional dimensionless ratios determine the relative importance of vibronic structure, including the detuning between electronic and vibrational scales and the effective vibronic coupling strength (e.g., γ/ω or an equivalent Huang–Rhys-type measure), which set how strongly the discrete mode participates in transfer. Because the three-regime narrative reported here—(i) effectively Gaussian/weakly anharmonic behavior at large Λ, (ii) intermittency-dominated behavior at Λ ≲ 1, and (iii) reduced sensitivity to bath statistics in the strong-mixing limit at large Δ—is expressed in terms of (η_NM_,Λ) and the electronic mixing scale Δ, it is not restricted to a single biochemical complex. Rather, it is expected to generalize to any donor–acceptor ET setting where (a) the environment exhibits finite memory (η_NM_ ≳ 1) and (b) environmental fluctuations include intermittent, discrete events that can be modeled as shot noise with rate λ_*p*_. Under these conditions, harmonic-bath treatments capture dynamics accurately in the frequent-small-event limit (Λ ≫ 1) but can fail in the sparse-event regime (Λ ≪ 1), where higher-order statistics and intermittency qualitatively modify population and coherence dynamics even under variance-matched conditions.

Memory effects are expected to be relevant in many biomolecular ET settings, where characteristic electronic tunneling scales (*ℏ*Δ) can be comparable to, or exceed, the dominant environmental frequency scales (e.g., bath cutoffs or relaxation rates), so that bath correlation times are not negligible on the system time scale and a non-Markovian treatment is required.^44^ In such regimes, Markovian approximations can break down and may yield unphysical artifacts (including violations of positivity or spurious population behavior) when applied outside their domain of validity, especially at stronger coupling.^45,46^ The non-Markovian NMSSE framework employed here avoids these issues by explicitly incorporating finite bath memory through the correlation kernel and its associated time-dependent coefficients.^47^ To define the environmental correlation function α_*G*_(*t*,*s*) in the harmonic (Gaussian) reference model, we specify a structured spectral density *J*(ω) composed of narrow bands that capture dominant vibrational features of the surrounding medium,^48^

28 $$J\left(\omega \right)=\underset{i}{\sum }\left(J_{i}\left(\omega \right)\right)$$

where

29 $$J_{i}\left(\omega \right)=\frac{\gamma \Gamma \omega_{i}^{2}\omega }{{\left(\omega_{i}^{2}-\omega^{2}\right)}^{2}+\Gamma^{2}\omega^{2}}$$

Here, γ is the coupling strength, ω_*i*_ are the dominant mode frequencies, and Γ is the bandwidth. Assuming white type noise and incorporating finite temperature *T* effects, the correlation function is given by^29^

30 $$\alpha_{G}\left(t,s\right)=\int_{0}^{\infty }d\omega J\left(\omega \right)\left[\coth\left(\frac{\omega }{2k_{B}T}\right)\left(\cos\left(\omega \left(t-s\right)\right)-i\sin\left(\omega \left(t-s\right)\right)\right)\right]$$

The NMQSD formulation systematically incorporates temperature into the coefficients *g*_*i*_(*t*), ensuring the model’s validity at experimental conditions (e.g., room temperature) and capturing the interplay between quantum coherence and thermal noise. The low-temperature condition *k*_B_ *T* ≪ *ℏ*ω places the system firmly in the quantum regime, wherein non-Markovian memory effects are pronounced. Experimental signatures can be accessed via, for example, 2D Raman spectroscopy or single-molecule fluorescence.^26,27^

For our particular system, the Lindblad operator representing the receptor’s coupling to the membrane is *L* = γ_*nj*_ σ_*z*_. Substituting this and our system Hamiltonian (eq 25) into the general NMSSE (eq 17) yields the final equation for our simulation:

31 $$\frac{d}{dt}{\mathrm{\psi ̃}}_{t}=-iH_{S}{\mathrm{\psi ̃}}_{t}+\gamma_{nj}\left(\sigma_{z}-\left\langle \sigma_{z}\right\rangle \right){\mathrm{\psi ̃}}_{t}{\mathrm{Z̃}}_{t}-g_{0}\left(t\right)\gamma_{nj}^{2}\left[\left(\sigma_{z}-\left\langle \sigma_{z}\right\rangle \right)\sigma_{z}-\left\langle \sigma_{z}-\left\langle \sigma_{z}\right\rangle \right\rangle \right]{\mathrm{\psi ̃}}_{t}+ig_{1}\left(t\right)\gamma_{nj}^{2}\Delta \left[\left(\sigma_{z}-\left\langle \sigma_{z}\right\rangle \right)\sigma_{y}-2\left\langle \sigma_{z}-\left\langle \sigma_{z}\right\rangle \sigma_{y}\right\rangle \right]{\mathrm{\psi ̃}}_{t}$$

where *z̃*_*t*_ is the shifted noise from equation­(eq 13).

By simulating NMSSE (eq 31) and calculating the density matrix via an ensemble average from eq 11, we determine the electron transfer dynamics. We quantify the influence of the vibrational modes by calculating the difference in maximum transfer probability with and without the protein’s vibrational coupling:

32 $$\Delta P=\mathrm{Max}[P_{D\to A}\left(t\right){]}_{with}-\mathrm{Max}[P_{D\to A}\left(t\right){]}_{without}$$

Furthermore, the time-dependent vibrational energy transfer from the donor to the acceptor site is calculated from

33 $$\left\langle n_{\mathrm{vib}}\left(t\right)\right\rangle =\left\langle \psi \left(t\right)\right|\mathrm{n̂}|\psi \left(t\right)$$

## 3 Results and Discussion

We evaluated donor → acceptor electron-transfer (ET) dynamics over a broad range of model parameters and donor–acceptor couplings by solving the NMSSE (eq 31) for both harmonic (Gaussian) and anharmonic, non-Gaussian (Poisson/shot-noise) environments. From ensembles of stochastic trajectories we constructed the reduced density matrix (eq 11) and extracted the transfer probability *P*_*D*→*A*_(*t*). To isolate the role of the discrete vibrational coordinate, we compare simulations performed with and without vibronic coupling and define Δ*P* = max_*t*_[*P*_*D*→*A*_(*t*)]_with vib_ – max_*t*_[*P*_*D*→*A*_(*t*)]_without vib_. Two-dimensional maps of Δ*P* therefore quantify when the discrete mode enhances ET and how that enhancement depends on bath memory and noise statistics.

Parameter choices were guided by prior biologically motivated vibration-assisted tunneling models, including olfactory-receptor-inspired settings,^13,49^ and are summarized in Table 1. In the structured-bath simulations, environmental memory is set by the bath correlation time τ_*c*_ (controlled by the line width Γ of the structured spectral density), and the degree of non-Gaussian intermittency is controlled by the event-per-memory parameter Λ≡λ_*p*_τ_*c*_ (mean number of events occurring within one correlation time). We use Λ ≫ 1 to denote the *weakly anharmonic* (frequent-small-event) regime, Λ ∼ 1 the intermediate regime, and Λ ≪ 1 the *strongly anharmonic* (rare, impulsive) regime. When scanning Λ ∈ {0.1,1,10} we calibrate the shot-noise variance to match the Gaussian bath at the level of the second cumulant, so that observed deviations primarily reflect higher-order non-Gaussian statistics rather than a trivial rescaling of noise strength. The electronic coupling is varied over Δ∈ {10^–4^,10^–3^,10^–2^,10^–1^} eV to probe weak-, intermediate-, and strong-mixing regimes.

**Table 1:** Parameters for the Numerical Simulations

ϵ_*A*_ – ϵ_*D*_ (cm^–1^)	Δ (eV)	γ (eV)	γ_*nj*_	ω_1_ (cm^–1^)	ω_2_ (cm^–1^)	ω_3_ (cm^–1^)	ω_4_ (cm^–1^)
500–1700	0.0001–0.1	0–0.49	0.005–0.05	836	1000	1240	1600

We first summarize the global impact of vibronic coupling and bath statistics using the ET-enhancement maps in Figure 2. In the weak-coupling limit (panels (a–b), Δ = 10^–4^ eV), ET is most sensitive to environmental fluctuations; accordingly, differences between Gaussian and Poisson/shot-noise descriptions are most visible and the non-Gaussian bath can yield a larger maximal transfer probability even under variance-matched conditions. In the strong-coupling limit (panels (c–d), Δ = 0.1 eV), coherent donor–acceptor mixing becomes dominant and the Gaussian and non-Gaussian predictions approach one another, indicating reduced sensitivity to the detailed noise statistics.

**Figure 2:**
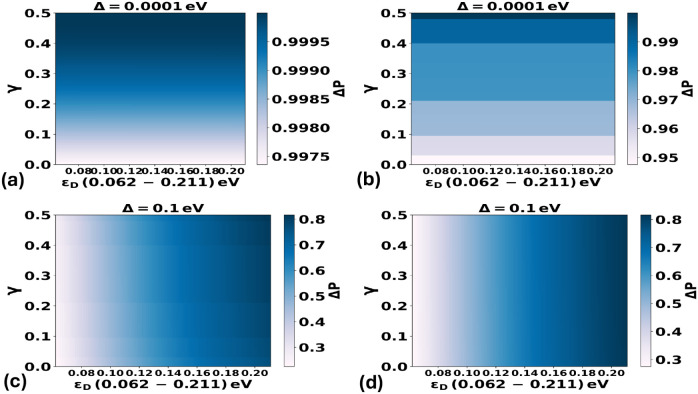
Comparison of the influence of a discrete vibrational mode on electron-transfer (ET) probabilities under harmonic (Gaussian) versus anharmonic, non-Gaussian (Poisson/shot-noise) environmental fluctuations across different donor–acceptor coupling strengths. Panels (a) and (b) show the comparison at the weakest electronic coupling, while panels (c) and (d) present the results at the strongest coupling. Panels (a–b) illustrate that at very small coupling (Δ = 0.0001 eV) the Poisson/shot-noise description can yield a higher maximum transfer probability than the Gaussian model even in the *weakly anharmonic* (frequent-small-event) regime at finite Λ (i.e., large but not asymptotically infinite). This residual difference decreases systematically as Λ is increased further, consistent with the expectation that a compound-Poisson bath approaches an effectively Gaussian bath when many small events occur within one correlation time. In contrast, panels (c–d) show that at strong coupling (Δ = 0.1 eV) the ET probabilities predicted by Gaussian and Poisson descriptions converge, indicating reduced sensitivity to noise statistics in the strong-coupling regime.

The qualitative trend is consistent with a simple physical picture: when Δ is small, transfer requires environmental assistance and is therefore sensitive to intermittency and rare events; when Δ is large, the system mixes strongly on its own and bath statistics mainly modulate (rather than determine) the transfer.

We next analyze the full time-dependent population dynamics to connect the regime map to trajectory-resolved dynamics.

Figure 3 shows donor/acceptor population dynamics in the frequent-small-event limit (Λ ≫ 1), where the compound-Poisson bath becomes effectively Gaussian by central-limit behavior. In this regime, harmonic (Gaussian) and Poisson/shot-noise predictions remain quantitatively close across the coupling range Δ∈ [10^–4^,10^–1^] eV, consistent with the expectation that harmonic-bath modeling is adequate when many small events occur within one correlation time. Residual differences at the smallest Δ can arise from intermittent jump realizations at the trajectory level, but these diminish as Λ increases further toward the Gaussianization limit.

**Figure 3:**
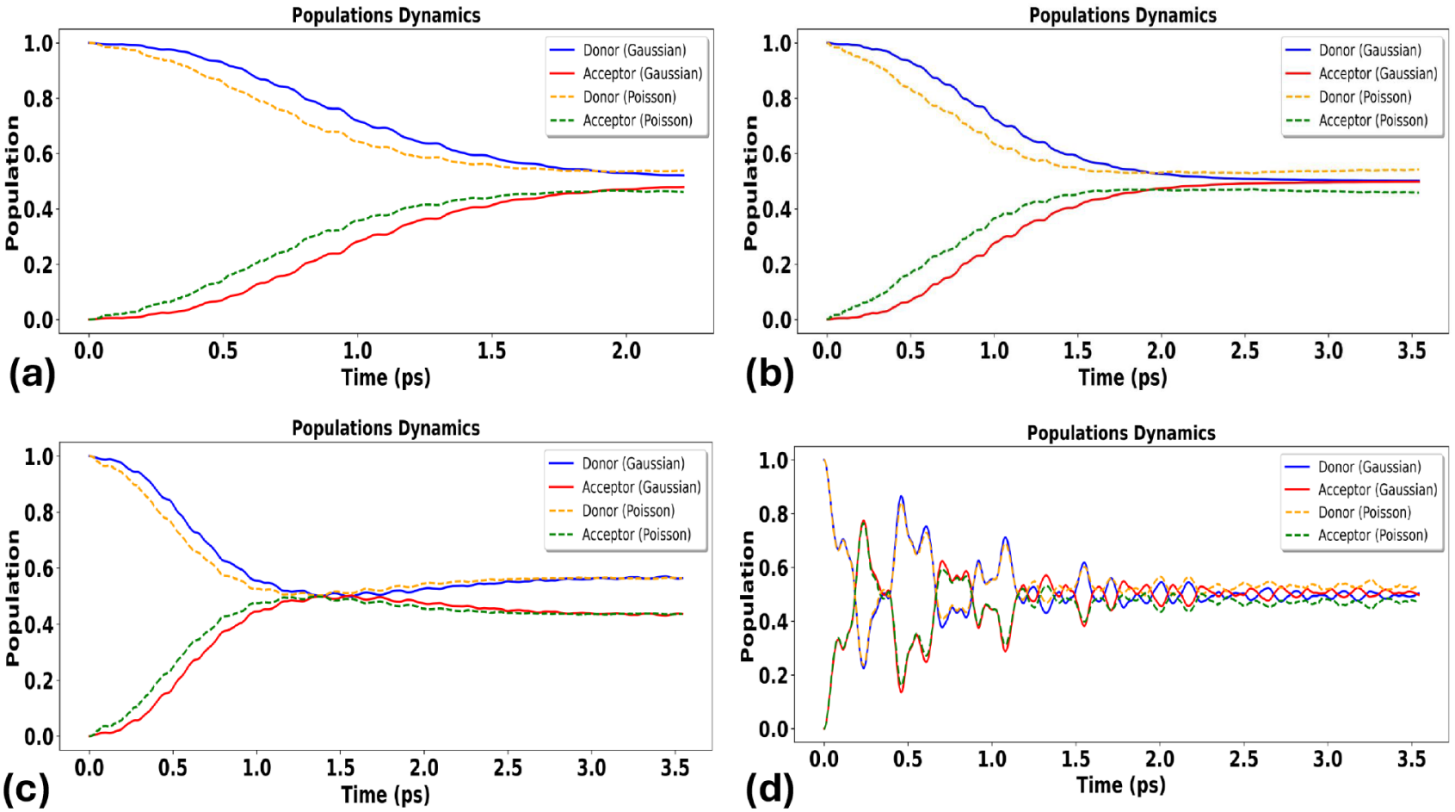
Comparison of the influence of a discrete vibrational mode on electron-transfer (ET) probabilities under harmonic (Gaussian) versus anharmonic, non-Gaussian (Poisson/shot-noise) environmental fluctuations across different donor–acceptor coupling strengths. Panels (a) and (b) show the comparison at the weakest electronic coupling, while panels (c) and (d) present the results at the strongest coupling. Panels (a–b) demonstrate that even in the weakly anharmonic regime and at very small coupling (Δ = 0.0001 eV), Poisson-driven intermittency can yield a noticeably higher transfer probability than the Gaussian model. In contrast, panels (c–d) show that at strong coupling (Δ = 0.1 eV) the ET probabilities predicted by Gaussian and Poisson descriptions converge, indicating reduced sensitivity to noise statistics in the strong-coupling regime.

Figure 4 presents population dynamics at intermediate intermittency (Λ ∼ 1). Here, departures between Gaussian and Poisson/shot-noise baths become more pronounced: intermittent events alter transient population flow and can enhance transfer relative to the harmonic baseline. The effect is again strongest at small Δ, where coherent mixing alone is insufficient to drive efficient transfer, and decreases with increasing Δ as coherent donor–acceptor mixing becomes more dominant.

**Figure 4:**
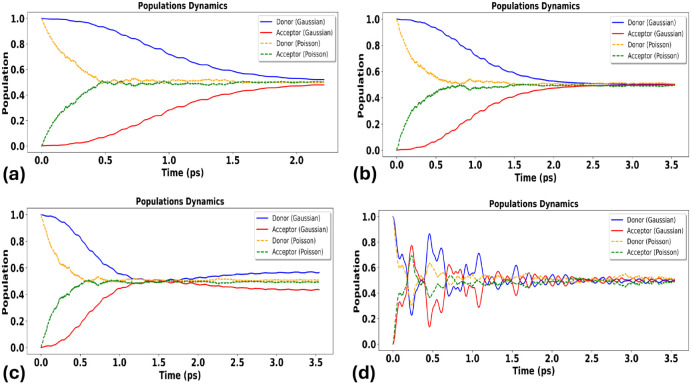
Population dynamics of donor and acceptor states in the donor–acceptor dimer under *intermediate anharmonicity*, computed with the non-Markovian stochastic Schrödinger equation (NMSSE) for harmonic (Gaussian) versus anharmonic, non-Gaussian (Poisson/shot-noise) environments. Panels (a–d) show increasing electronic coupling Δ: (a) Δ = 0.0001 eV; (b) Δ = 0.001 eV; (c) Δ = 0.01 eV; (d) Δ = 0.1 eV.

In the sparse-event limit (Λ ≪ 1), Figure 5 shows that population dynamics can deviate qualitatively from the harmonic prediction across essentially all couplings. Rare, impulsive events produce abrupt changes in the effective system driving and therefore reshape transient dynamics and the maximum transfer probability. Even in this regime, however, increasing Δ*r*educes sensitivity to bath statistics: at strong coupling (Δ = 0.1 eV) the harmonic and non-Gaussian predictions move closer, consistent with a noise-insensitive strong-mixing limit.

**Figure 5:**
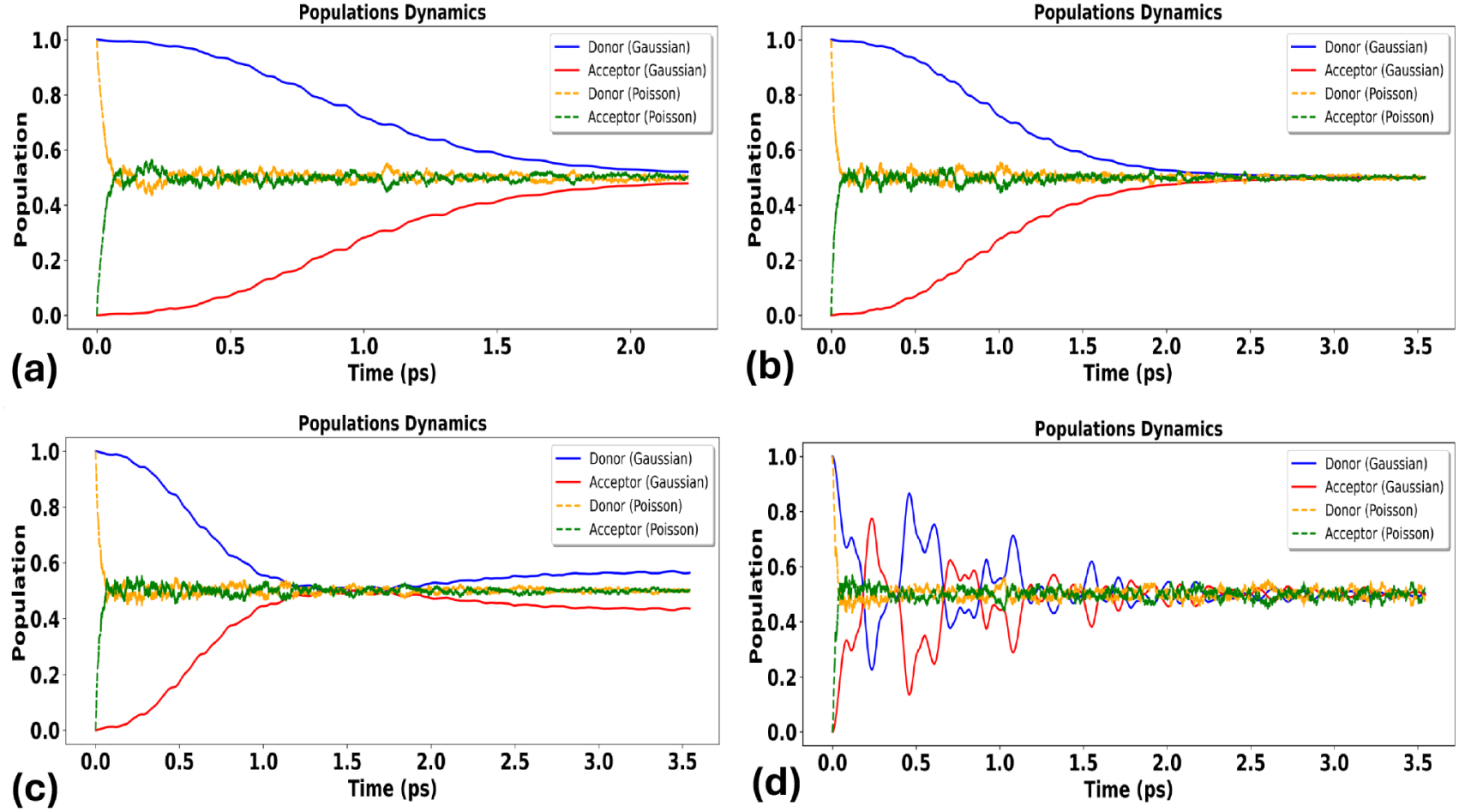
Population dynamics of donor and acceptor states under *strong anharmonicity*, computed via the non-Markovian stochastic Schrödinger equation (NMSSE) for harmonic (Gaussian) versus anharmonic, non-Gaussian (Poisson/shot-noise) environments. Panels (a–d) show increasing donor–acceptor coupling Δ: (a) Δ = 0.0001 eV; (b) Δ = 0.001 eV; (c) Δ = 0.01 eV; (d) Δ = 0.1 eV.

To directly probe how bath statistics reshape coupled electron–vibration dynamics, we compute the time-dependent vibrational energy of the discrete mode from ensemble-averaged trajectories. Figure 6 shows that increasing anharmonicity/intermittency produces increasingly irregular and larger-amplitude vibrational-energy fluctuations. In the weakly anharmonic regime (Figure 6a, Λ ≫ 1), energy flow is comparatively regular and close to the harmonic-bath baseline. In the intermediate regime (Figure 6b, Λ ∼ 1), distortions become clearly visible, indicating modified energy exchange during transfer. In the strongly anharmonic regime (Figure 6c, Λ ≪ 1), sparse events drive pronounced, irregular energy excursions, consistent with strong intermittent perturbations of the coupled electronic–vibrational manifold. These trends support the interpretation that Λ = λ_*p*_τ_*c*_ is a decisive control parameter not only for ET efficiency but also for the accompanying vibrational energy flow.

**Figure 6:**
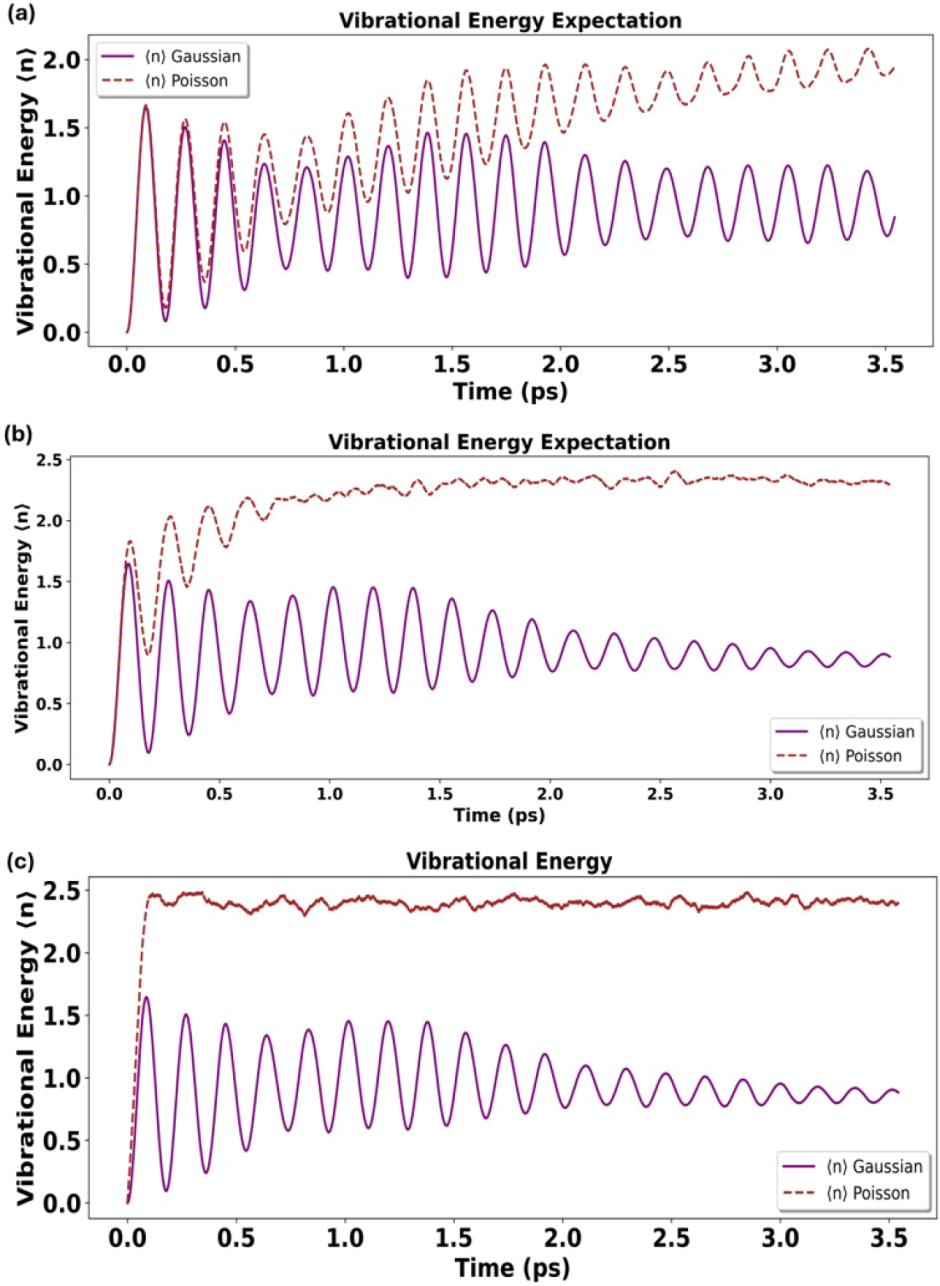
(a–c) Time evolution of the vibrational energy expectation value during donor–acceptor electron transfer for increasing environmental anharmonicity, computed from ensemble-averaged trajectories. (a) Weak anharmonicity (many small events per correlation time, Λ ≫ 1): near-harmonic energy exchange with only minor deviations from the Gaussian baseline. (b) Intermediate intermittency (Λ ∼ 1): non-Gaussian events produce measurable distortions in energy flow. (c) Strong anharmonicity (sparse events, Λ ≪ 1): large-amplitude, irregular fluctuations reflecting strong intermittent perturbations of the coupled electron–vibration dynamics.

While ET mechanisms in specific receptor–ligand complexes are system dependent and ultimately require experimental confirmation, Quantum Biological Electron Tunneling (QBET) spectroscopy provides a general route to probe ET dynamics in biomolecular settings. QBET enables optical detection of electron-transfer events in real time under biologically relevant conditions and has been demonstrated in redox-active proteins such as mitochondrial cytochrome *c*.^28^ In principle, analogous strategies could be applied to engineered donor–acceptor protein constructs or receptor–ligand complexes with suitable redox centers by coupling them to plasmonic or optical reporters. Time-resolved readouts could then test sensitivity to bath memory and intermittency, providing constraints for system-specific parametrization of τ_*c*_ and the event statistics underlying Λ.

## 4 Conclusions

We have presented a dynamical open-quantum-systems study of electron transfer (ET) in a minimal receptor–ligand donor–acceptor platform using a non-Markovian stochastic Schrödinger equation (NMSSE) framework. By contrasting the standard *harmonic* (Gaussian) bath approximation with an *anharmonic*, non-Gaussian description implemented through compound-Poisson (shot-noise) event statistics and jump-like perturbations, we clarified how environmental memory and fluctuation statistics regulate ET beyond Markovian or purely Gaussian assumptions. Our objective was methodological and general: we do not advance a mechanism for any specific biomolecular complex, but instead provide a controlled framework for assessing when harmonic-bath modeling is reliable and when it fails.

Across representative parameter ranges, three robust regimes emerged. (i) In the *weakly anharmonic* regime, characterized by many small events per correlation time (Λ = λ_*p*_τ_*c*_ ≫ 1), the compound-Poisson bath becomes *effectively* Gaussian and the harmonic and non-Gaussian predictions are quantitatively close. In the *weakly anharmonic* regime when Δ*b*ecome large, coherent donor–acceptor mixing dominates and sensitivity to the detailed noise statistics diminishes, leading to convergence between the Gaussian and non-Gaussian predictions. (ii) In the *intermediate anharmonic* regime (Λ ≲ 1), intermittency and higher-order non-Gaussian statistics become dynamically relevant, enhancing ET and qualitatively reshaping the time dependence of populations and coherences, particularly at weak electronic coupling. (iii) In the strongly anharmonic regime (Λ ≪ 1), sparse events drive pronounced, irregular energy excursions, consistent with strong intermittent perturbations of the coupled electronic–vibrational manifold. Together, these regimes provide practical guidance for when Gaussian (harmonic) approximations are adequate and when explicit modeling of anharmonic, non-Gaussian bath statistics is required for faithful ET dynamics in complex environments.

More broadly, our results indicate that ET can display a degree of resilience to environmental fluctuations, but that the *character* of the noise—diffusive versus intermittent—can still leave strong dynamical signatures. In particular, Poissonian intermittency can induce enhanced transfer or altered transient behavior even under variance-matched conditions, providing a route to isolate the impact of higher cumulants beyond the harmonic-bath paradigm.

Future work is motivated by two primary limitations. First, the parametrization employed here is representative rather than system-specific; for concrete complexes, structured spectral densities, memory times, and event statistics can be constrained using vibrational and ultrafast spectroscopies (e.g., Raman/2D techniques) and incorporated into the present framework. Second, experimental validation of non-Gaussian intermittency effects remains an open challenge. In general, time-resolved electron-transfer probes—including QBET-style optical approaches in suitably engineered redox-active protein/ligand constructs—could test the predicted sensitivity to event-rate regimes and bath memory. On the theory side, extensions to multimode structured environments, temperature-dependent non-Condon effects, and cross-validation against numerically exact methods (e.g., HOPS/HEOM in selected regimes) will further strengthen the predictive power of non-Markovian, non-Gaussian modeling. Overall, this work establishes a systematic basis for going beyond harmonic-bath approximations by delineating the parameter regimes in which anharmonic, jump-like environmental fluctuations substantially affect ET dynamics.

## Supplementary Material





## Data Availability

The data sets and parameter values utilized in this study are used from previously published sources as cited. Researchers seeking additional information or access to these data sets are encouraged to go to this https://github.com/waqashaseeb.

## Notes

Additional numerical results that complement the main text by reporting time-resolved coherence dynamics (off-diagonal reduced density-matrix elements) and vibrational excitation dynamics (PDF)
Tex (overleaf code) (ZIP)
