# Supplementary material for: Non-Markovian Electron Transfer in Ligand–Receptor Complexes: Insights from Non-Gaussian Anharmonic Baths
Source: J Phys Chem B. 2026 Apr 15;130(17):4517–27. doi: 10.1021/acs.jpcb.6c00165 (PMC13137252; doi:10.1021/acs.jpcb.6c00165)
Supplement: Supplementary file 1 [file jp6c00165_si_001.pdf]

Non-Markovian Electron Transfer in Ligand–Receptor Complexes: Insights from Non-Gaussian Anharmonic Baths

Muhammad Waqas Haseeb† and Mohamad Toutounji*,†

†*Department of Physics, United Arab Emirates University, Al-Ain, UAE*

‡*Department of Chemistry, United Arab Emirates University, Al-Ain, UAE*

E-mail: mtotounji@uaeu.ac.ae

Overview

This Supplementary Information provides additional numerical results that complement the main text by reporting time-resolved coherence dynamics (off-diagonal reduced density-matrix elements) and vibrational excitation dynamics (mode occupancy $\langle n(t) \rangle$) for the receptor–ligand donor–acceptor model studied in the manuscript. The simulations compare harmonic (Gaussian) environmental fluctuations against an anharmonic, non-Gaussian description implemented via compound-Poisson (shot-noise) events, across electronic couplings $\Delta = 10^{-4}$–10^{-1} eV and representative event-rate regimes. To connect with the terminology of the main text, we use the event-per-memory parameter $\Lambda = \lambda_p \tau_c$ (mean number of discrete events per bath correlation time): the frequent-small-event limit ($\Lambda \gg 1$) is effectively Gaussian and corresponds to *weakly anharmonic* conditions, whereas the sparse, impulsive limit ($\Lambda \ll 1$) is genuinely non-Gaussian and corresponds to *strongly anharmonic* conditions. In the present data we illustrate the frequent-small-event limit , while keeping the remaining

system and bath parameters fixed as in the main text. The coherence traces quantify how environmental statistics affect phase relationships and decoherence pathways, while $\langle n(t)\rangle$ highlights how energy exchange with the discrete vibrational mode depends on diffusive (Gaussian) versus intermittent (shot-noise) driving.

Coherence Dynamics

Figure S1(a–d) shows the coherence evolution of the reduced donor–acceptor subsystem under harmonic (Gaussian) versus non-Gaussian (compound-Poisson) environmental fluctuations in the *frequent-event* regime ($\lambda_p = 0.1$; weakly anharmonic, $\Lambda \gg 1$ for fixed τ_c), At the weakest coupling ($\Delta = 10^{-4}\,\mathrm{eV}$), coherent donor–acceptor mixing is minimal and the coherence remains weak, with intermittent jump events producing distinct transient features relative to the Gaussian baseline. As Δ increases to 10^{-3}–$10^{-2}\,\mathrm{eV}$, coherent mixing becomes more apparent and the coherence develops more pronounced oscillatory structure; in this regime, intermittency can modify the damping envelope and transient phase/amplitude compared to the Gaussian model under the same second-cumulant calibration. At the largest coupling ($\Delta = 10^{-1}\,\mathrm{eV}$), coherent mixing dominates and the relative differences between Gaussian and non-Gaussian coherence traces are reduced, consistent with the diminished sensitivity to bath statistics in the strong-coupling limit discussed in the main text.

Vibrational Energy Dynamics

To further characterize coupled electron–vibration dynamics, we compute the vibrational excitation of the discrete mode through the number operator $\hat{n} = a^\dagger a$ and report $\langle n(t)\rangle \equiv \langle\psi(t)|\hat{n}|\psi(t)\rangle$ as a vibrational-energy proxy. Figure S2(a–d) shows $\langle n(t)\rangle$ in the frequent-event (weakly anharmonic) regime, $\lambda_p = 0.1$, where the compound-Poisson bath is effectively Gaussian when many events occur within one memory time. In this regime, intermittent environmental events lead to energy exchange, producing irregular excursions and, in some

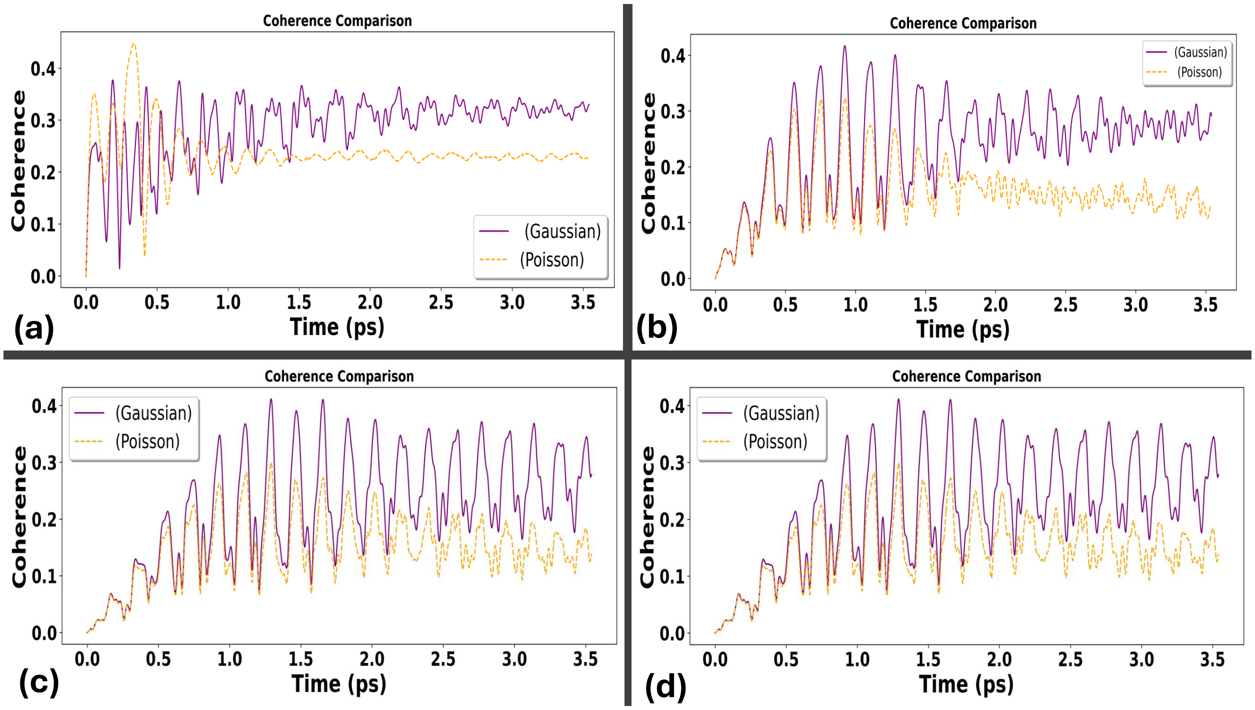

Figure S1: (a–d) Reduced donor–acceptor coherence dynamics under harmonic (Gaussian) versus non-Gaussian (compound-Poisson/shot-noise) environmental fluctuations in the sparse-event (strongly anharmonic) regime, $\lambda_p = 0.1$ (simulation units). The remaining parameters are as in the main text (including $\gamma_{nj} = 0.025\,\text{eV}$ and $\gamma_i = 0.1\,\text{eV}$). Panels correspond to increasing electronic coupling: (a) $\Delta = 0.0001\,\text{eV}$, (b) $\Delta = 0.001\,\text{eV}$, (c) $\Delta = 0.01\,\text{eV}$, and (d) $\Delta = 0.1\,\text{eV}$. In each panel, Gaussian (harmonic) and shot-noise (anharmonic) predictions are overlaid to highlight how non-Gaussian intermittency modifies coherence build-up and decay across coupling regimes.

cases, elevated long-time excitation relative to the harmonic (Gaussian) baseline, depending on the donor–acceptor coupling Δ. As Δ increases, coherent donor–acceptor mixing more strongly drives the vibrational coordinate and the oscillatory structure in $\langle n(t) \rangle$ becomes more pronounced; the extent to which non-Gaussian intermittency modifies the transient envelope or any long-time plateau is therefore Δ-dependent, with reduced sensitivity expected in the strongest-coupling limit where coherent dynamics dominates.

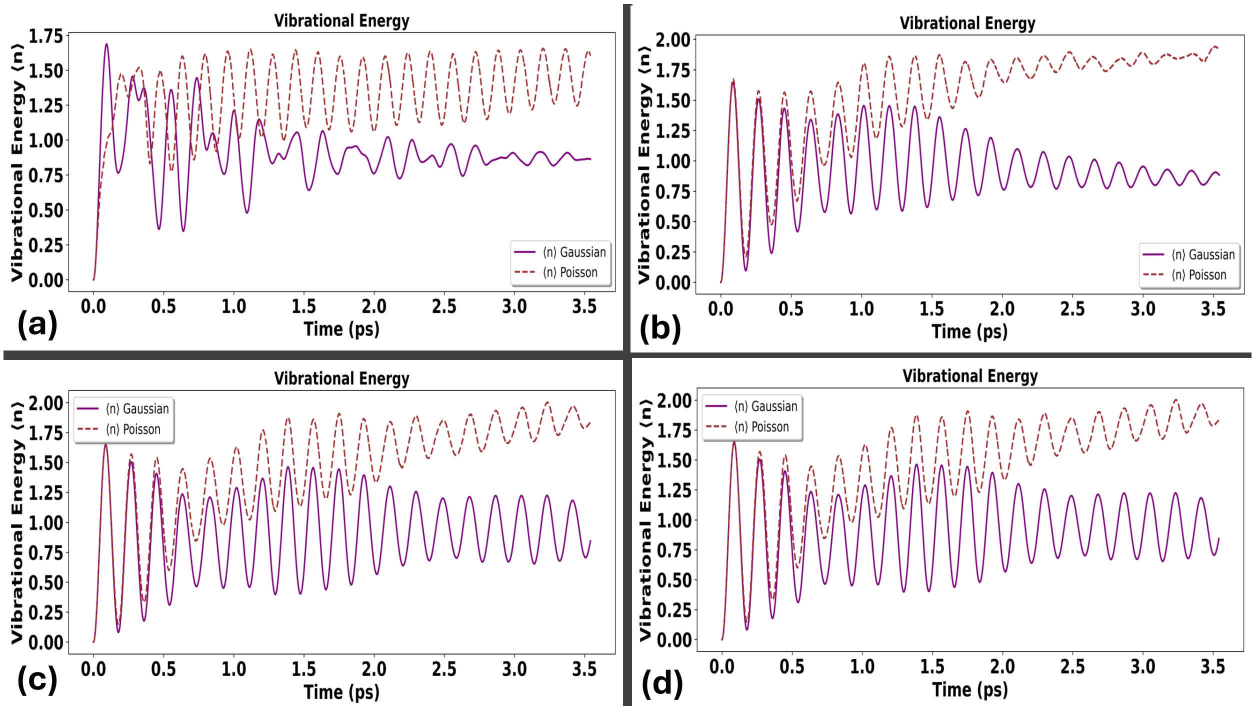

Figure S2: (a–d) Vibrational excitation dynamics $\langle n(t) \rangle$ under harmonic (Gaussian) versus non-Gaussian (compound-Poisson/shot-noise) environmental fluctuations in the frequent-event (weakly anharmonic) regime, $\lambda_p = 0.1$ (simulation units). Parameters are as in the main text (including $\gamma_{nj} = 0.025\,\text{eV}$ and $\gamma_i = 0.1\,\text{eV}$). Panels correspond to increasing electronic coupling: (a) $\Delta = 0.0001\,\text{eV}$, (b) $\Delta = 0.001\,\text{eV}$, (c) $\Delta = 0.01\,\text{eV}$, and (d) $\Delta = 0.1\,\text{eV}$. The comparison highlights how sparse, impulsive fluctuations can modify vibrational energy uptake and retention compared to the harmonic-bath baseline.
