# Supplementary figures and images for: Non-Markovian Electron Transfer in Ligand–Receptor Complexes: Insights from Non-Gaussian Anharmonic Baths
Source: J Phys Chem B. 2026 Apr 15;130(17):4517–27. doi: 10.1021/acs.jpcb.6c00165 (PMC13137252; doi:10.1021/acs.jpcb.6c00165)

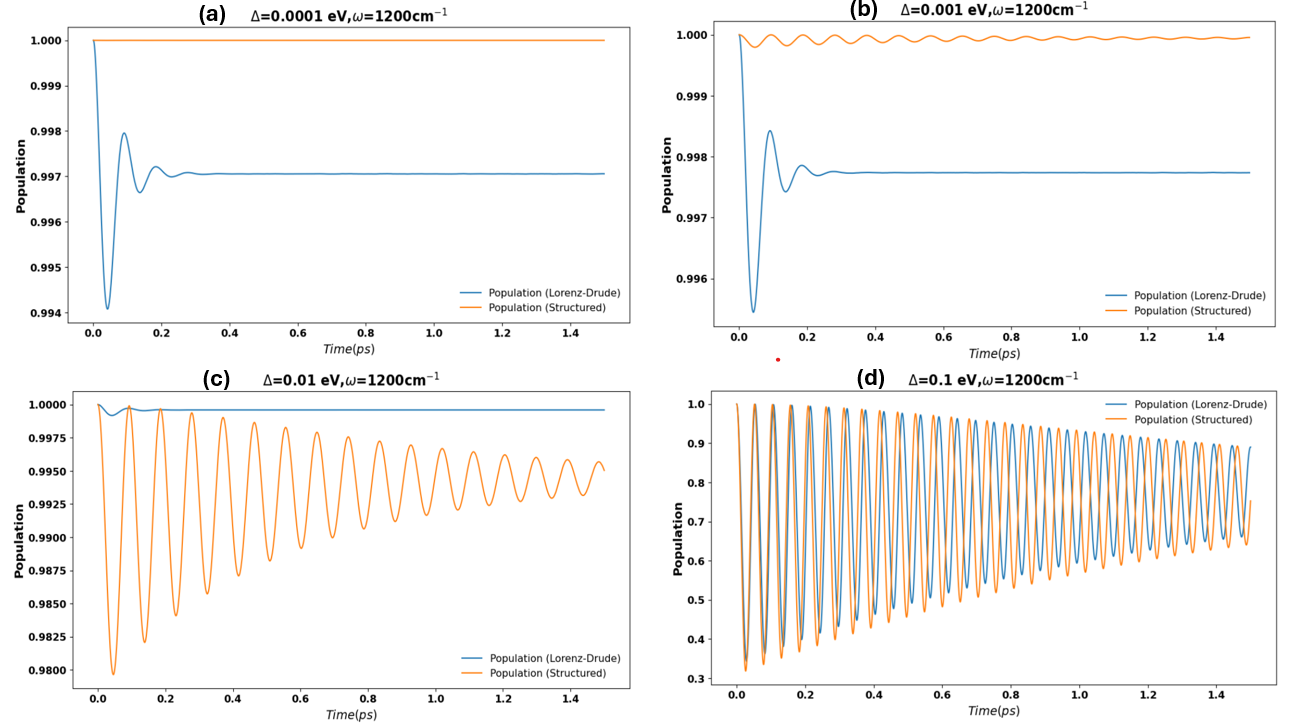

Supplement: Supplementary file 2 [file jp6c00165_si_002.zip › combined papulation 1200cm.png]

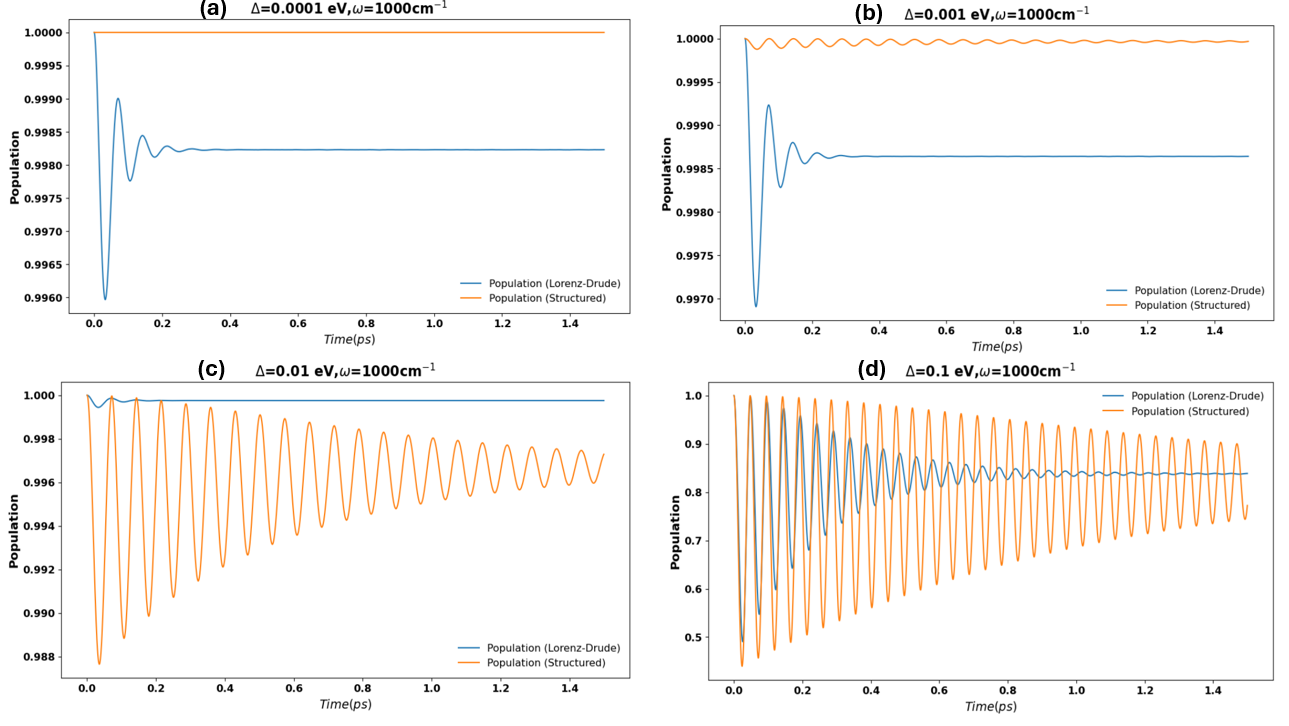

Supplement: Supplementary file 2 [file jp6c00165_si_002.zip › combined papulation 1000cm.png]

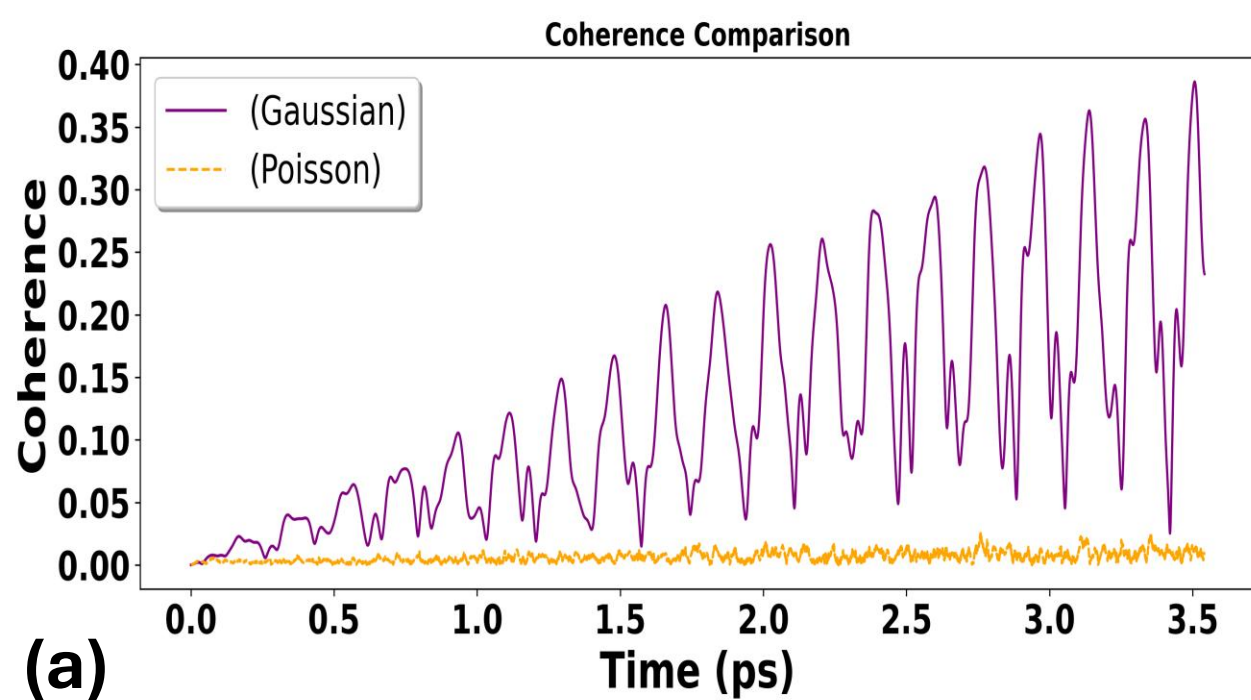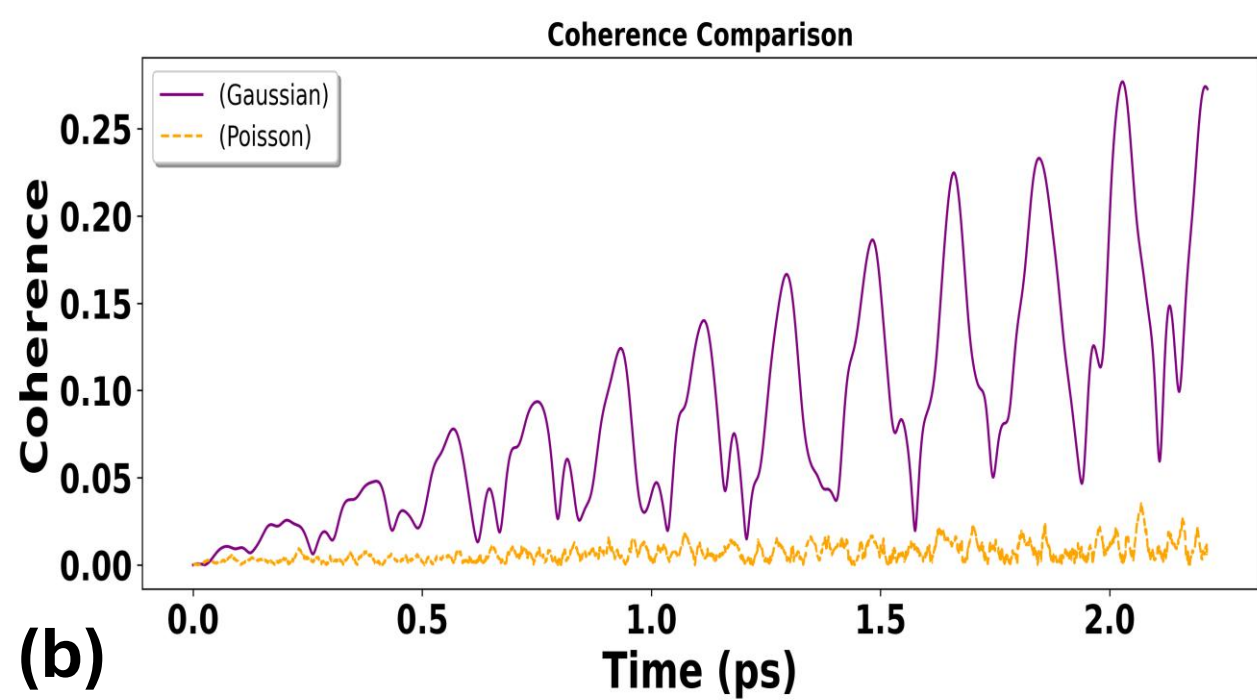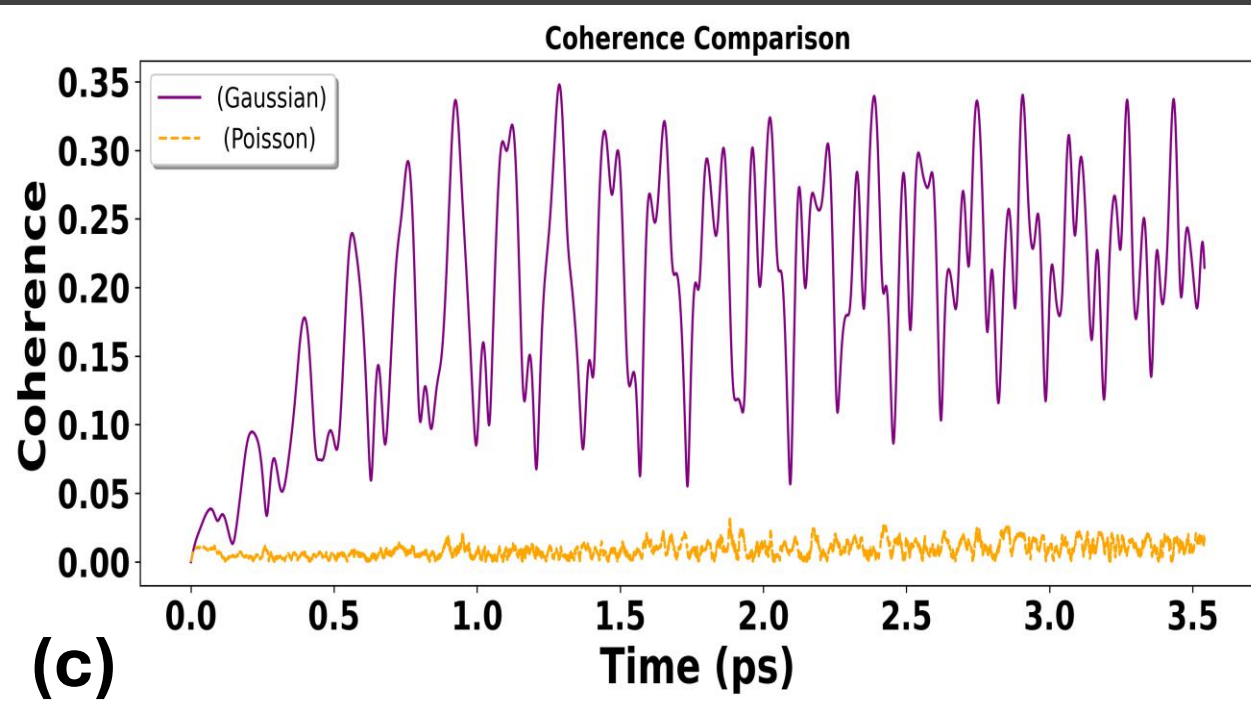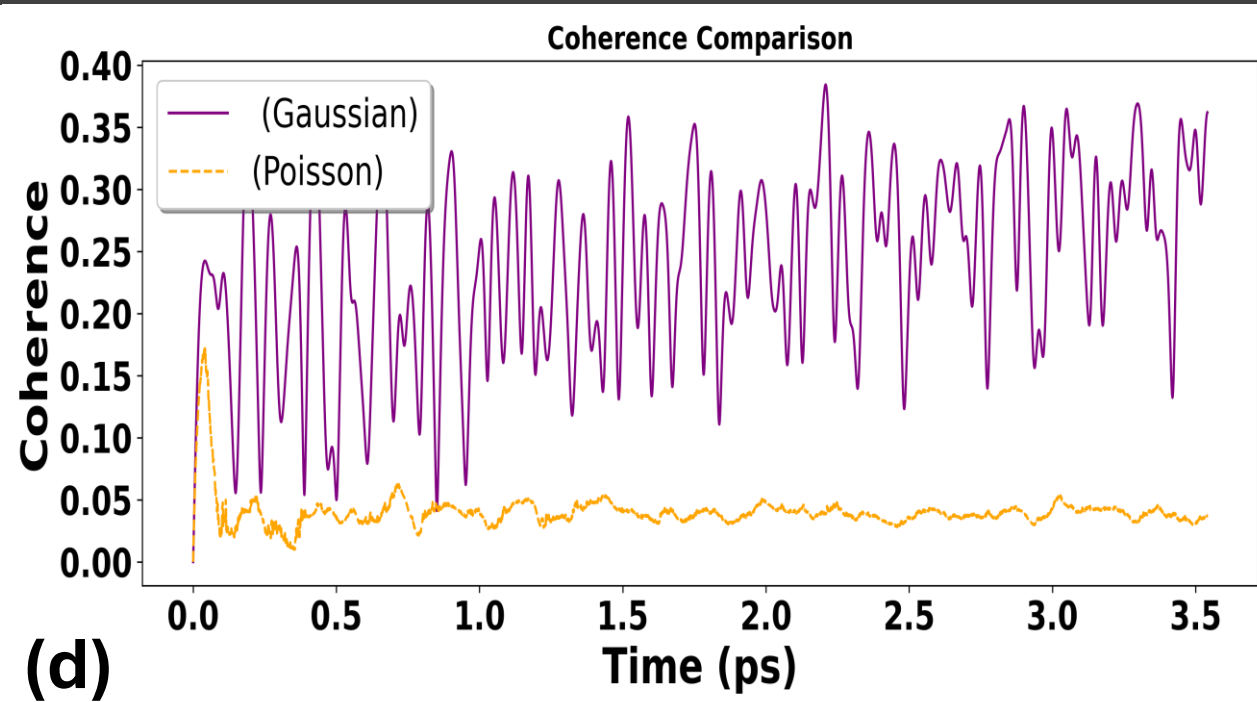

Supplement: Supplementary file 2 [file jp6c00165_si_002.zip › Figure_7.pdf]

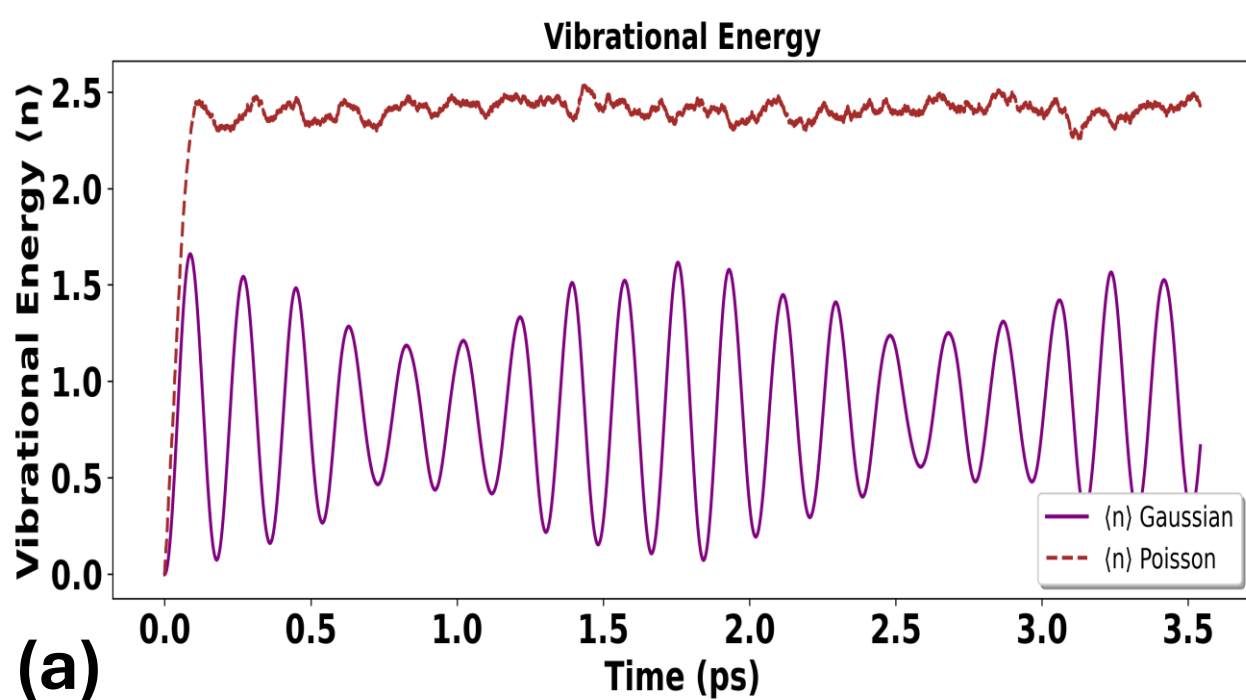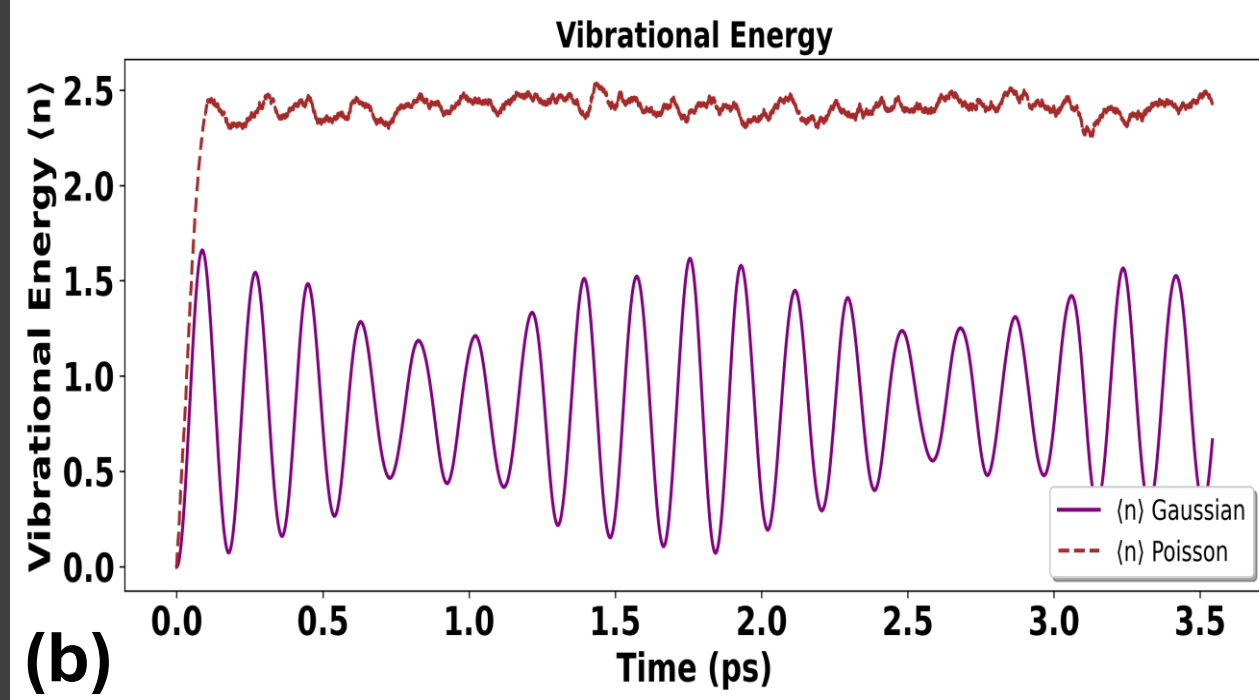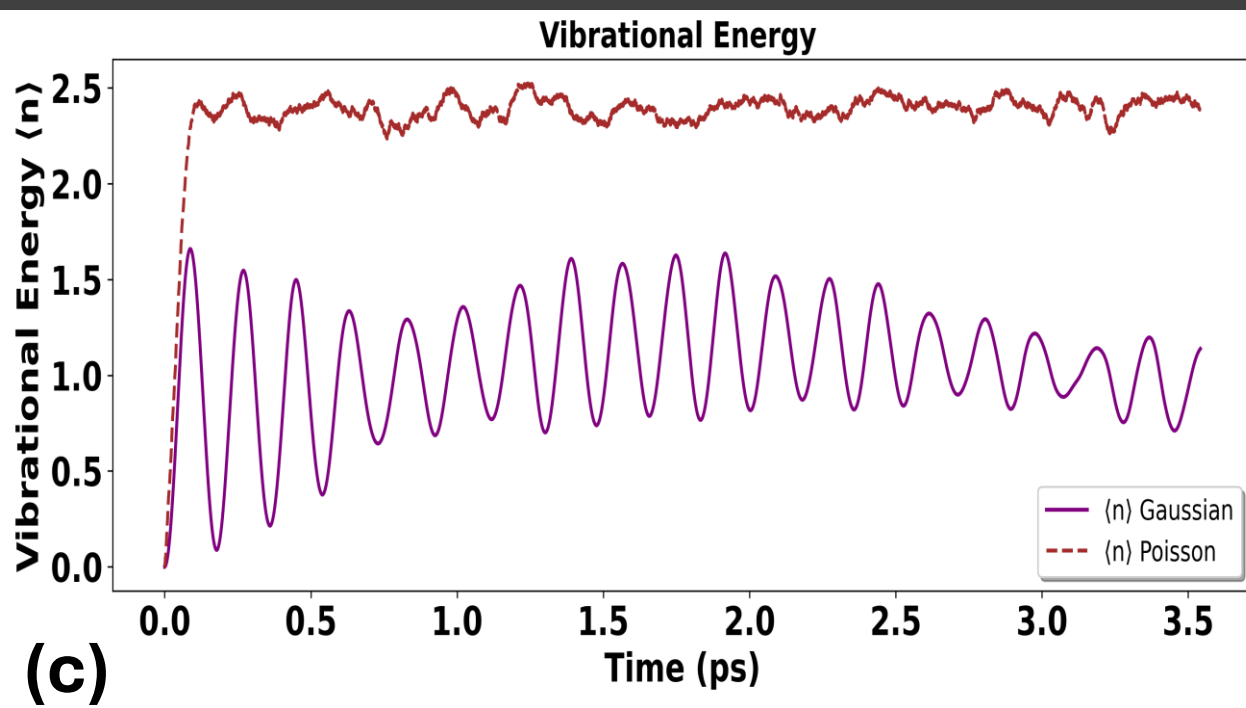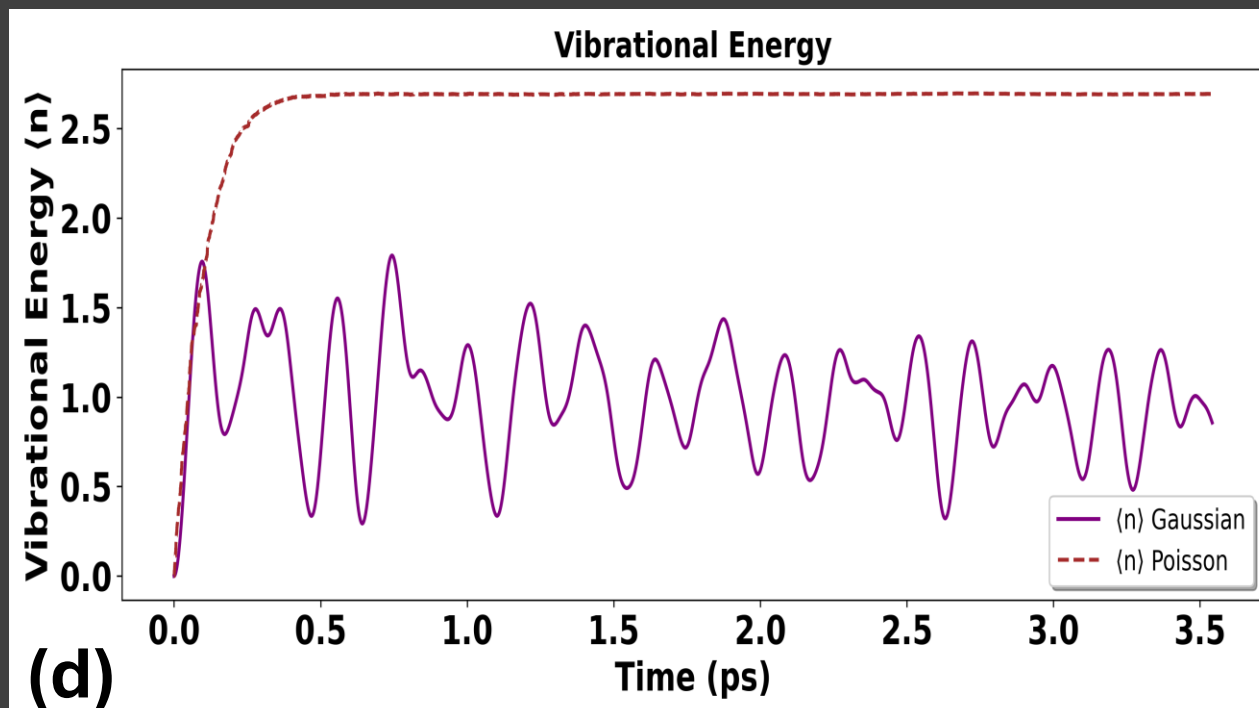

Supplement: Supplementary file 2 [file jp6c00165_si_002.zip › Figure_10.pdf]

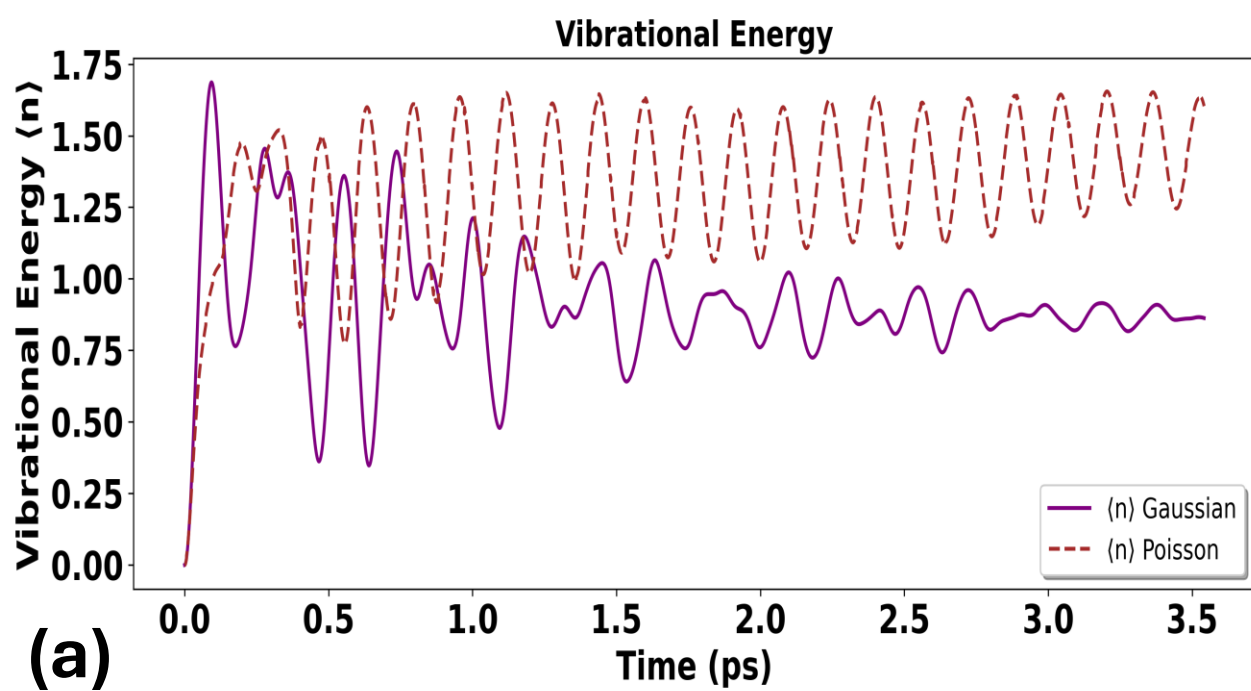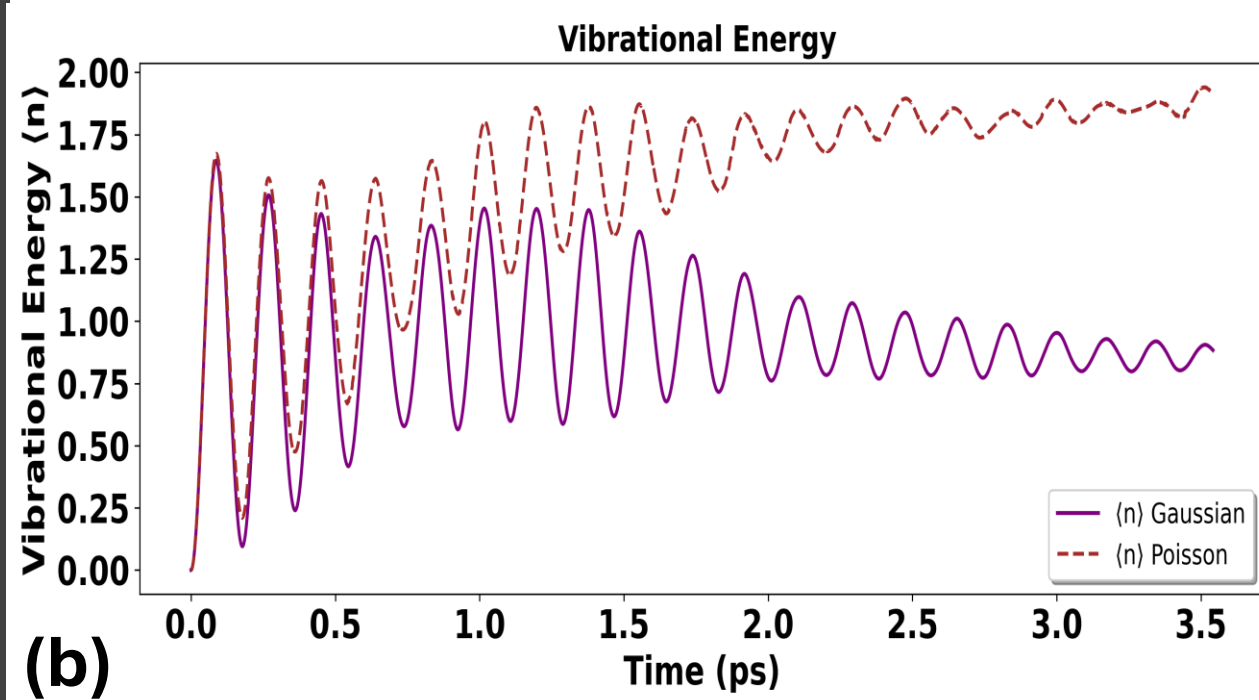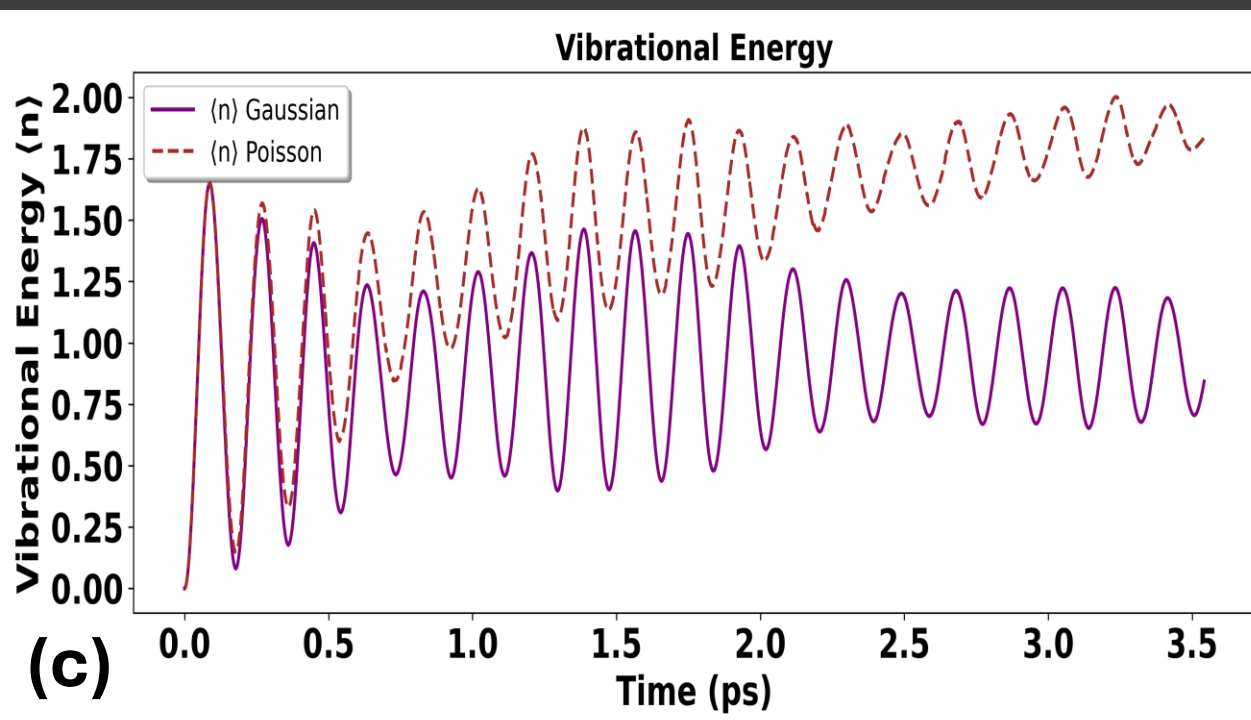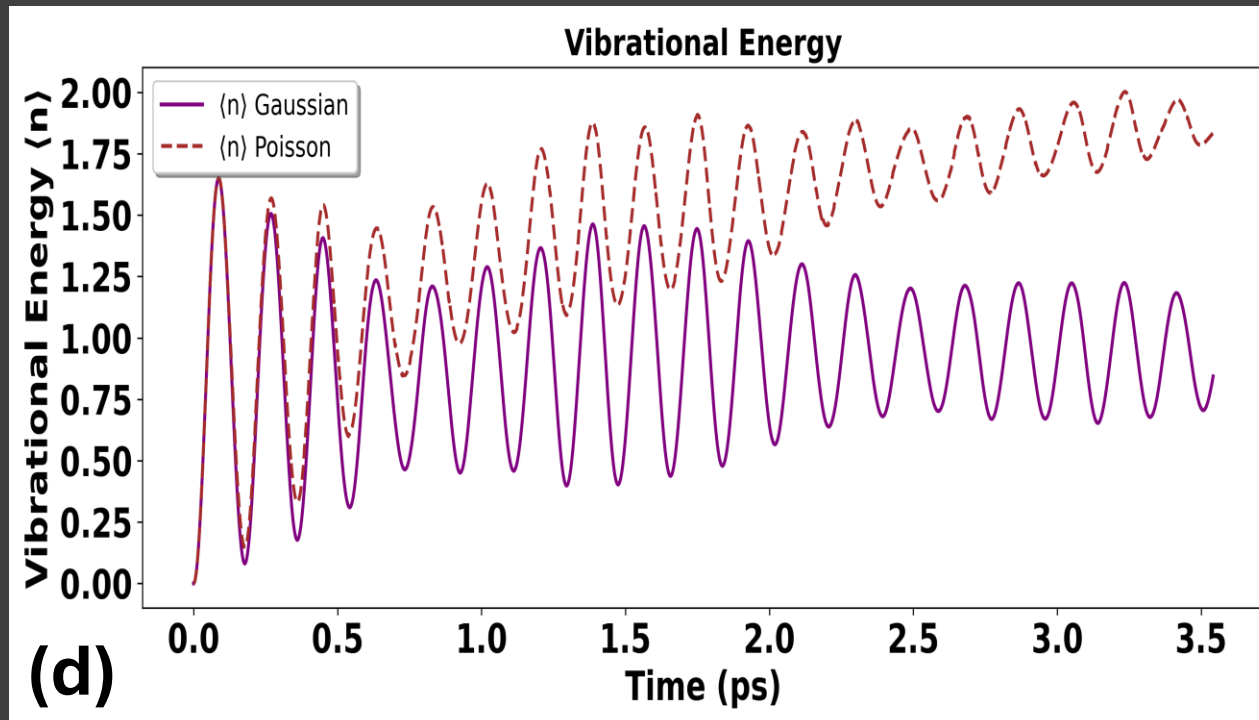

Supplement: Supplementary file 2 [file jp6c00165_si_002.zip › Figure_9.pdf]

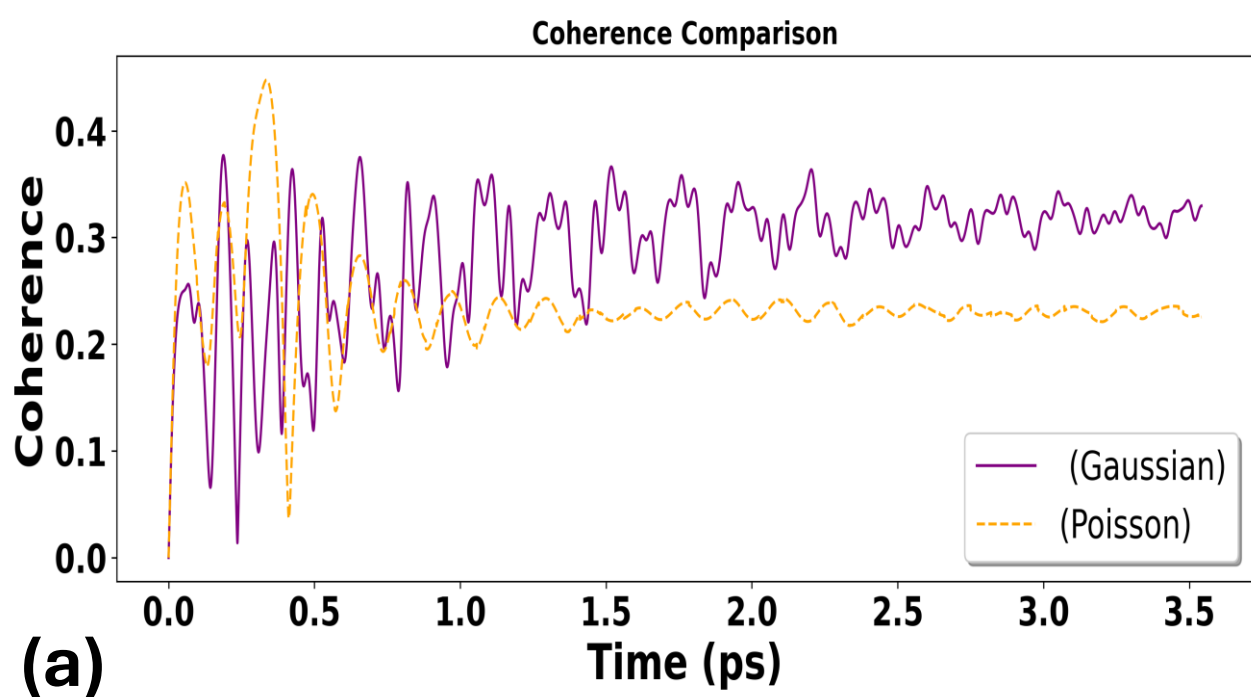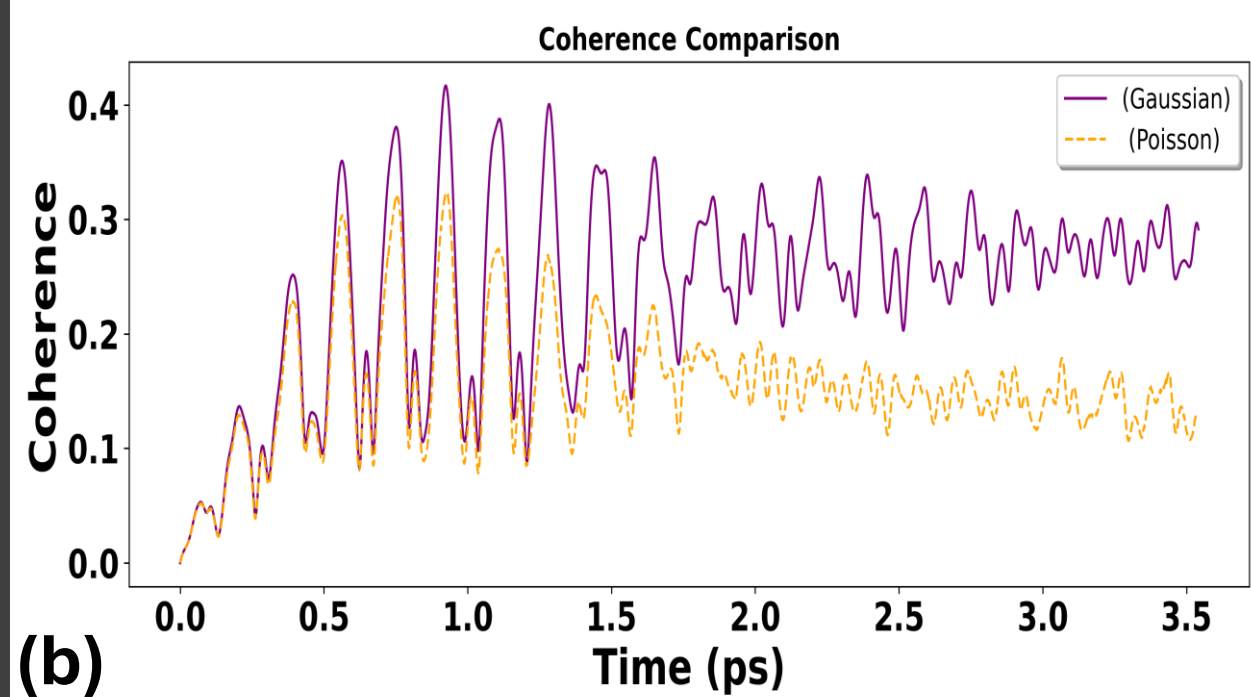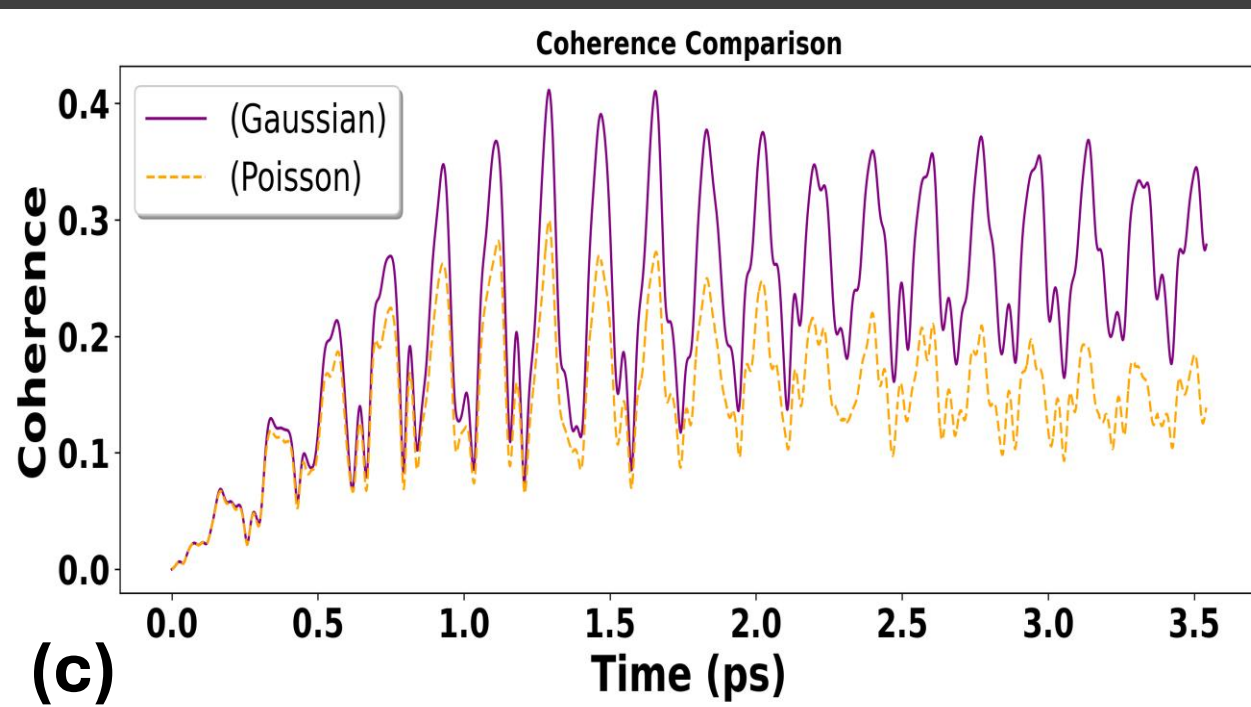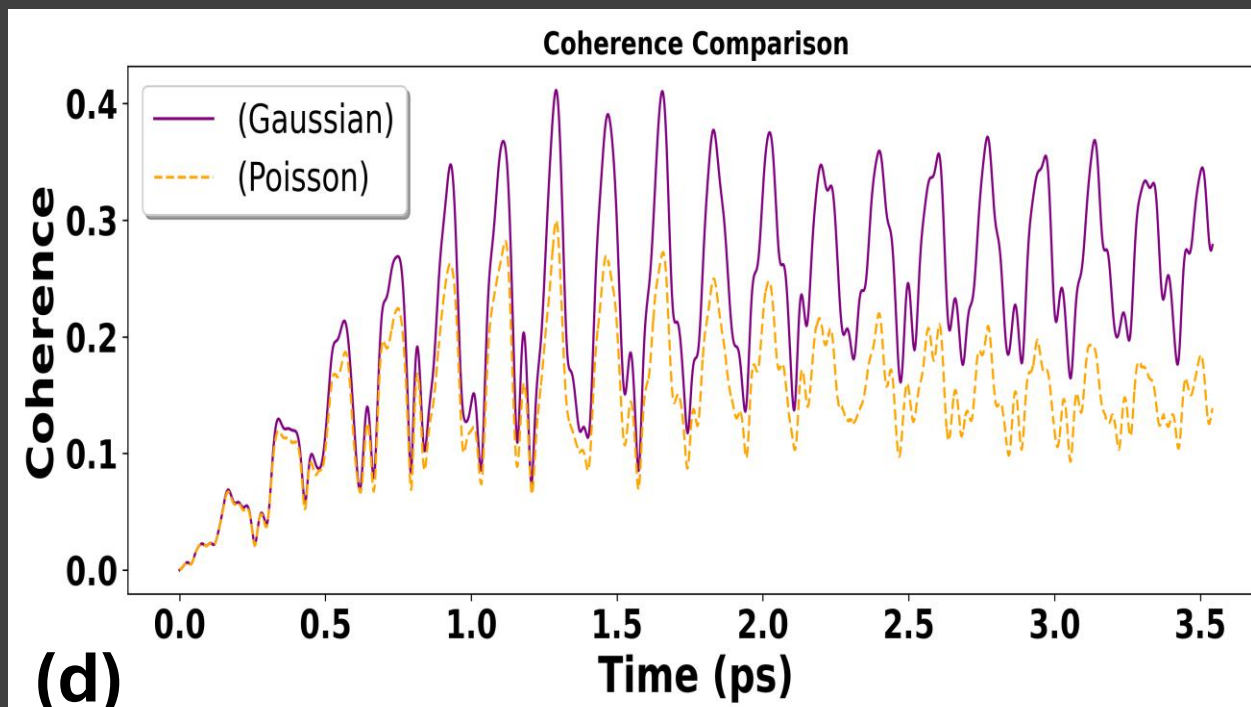

Supplement: Supplementary file 2 [file jp6c00165_si_002.zip › Figure_8_SI.pdf]
